# Signalling Entropy Across Measurement Scales: A Compositional Dilution Lemma and Cross-Modality Invariance for Information-Theoretic Analysis of Cancer Transcriptomes

**DOI:** 10.3390/e28070781

**Published:** 2026-07-09

**Authors:** Ömer Akgüller, Mehmet Ali Balcı, Ceren Uçmakoğlu, Lucian Gaban

**Affiliations:** 1 Department of Mathematics, Faculty of Science, Mugla Sitki Kocman University, 48000 Mugla, Türkiye; oakguller@mu.edu.tr (Ö.A.); cerenucmakoglu@posta.mu.edu.tr (C.U.); 2 Department of Oncology, Institute of Health Sciences, Dokuz Eylul University, 35340 Izmir, Türkiye; 3 Faculty of Economics, “1 Decembrie 1918” University of Alba Iulia, 510009 Alba Iulia, Romania

**Keywords:** signalling entropy, information theory, cancer transcriptomics, single-cell RNA sequencing, cross-modality validation, mixed-effects regression, protein–protein interaction networks, total correlation, 62P10, 92C42, 94A17, 92D20, 62J05

## Abstract

We develop a unified information-theoretic framework for the analysis of cancer transcriptomic dysregulation across measurement modalities. Three functionals capture distributional, network-aware, and joint-dependence aspects of expression: the Shannon entropy with a Miller–Madow correction, the signalling entropy rate over the protein interaction graph, and the Gaussian total correlation on a principal-component projection. A closed-form algebraic expression yields a linear-time algorithm for the signalling entropy rate. A Compositional Dilution Lemma decomposes bulk entropy into intrinsic and compositional contributions, and a Cross-Modality Invariance Proposition provides an empirically falsifiable null hypothesis. Validation uses 700,202 single cells and 3942 bulk samples across five cancer types. Pan-cancer tumour elevation is significant at $p<10^{-7}$, and cross-modality testing on 4230 observations does not reject the interaction null at p>0.5. The invariance conclusion is corroborated by cancer-level paired sign-flip permutation, cancer-block bootstrap, and empirical distribution function tests, and the prognostic Cox regressions satisfy proportional-hazards diagnostics with cross-validation concordance of 0.696±0.018. Immune deconvolution against the LM22 signature validates cell-type-specific predictions, partitioning cancers into myeloid-driven and lymphoid-driven classes. Breast cancer Cox regressions instantiate the predicted orthogonality of distributional and network-aware functionals after immune adjustment.

## 1. Introduction

Cancer is a disease of dysregulated information processing. The transformation of a normal cell into a malignant one involves the disruption of the regulatory programs that maintain tissue-specific gene expression, the rewiring of intracellular signalling networks, and the destabilisation of population-level coordination among neighbouring cells [1,2]. These processes leave systematic, quantifiable fingerprints in transcriptomic measurements, and the analysis of such fingerprints across high-throughput sequencing modalities has become a central methodological problem in computational systems biology. The challenge that motivates the present work lies in the construction of analytical frameworks that capture these fingerprints in a manner that is simultaneously theoretically grounded, computationally scalable, and consistent across the increasingly diverse set of measurement modalities through which cancer transcriptomes are now profiled.

Information-theoretic functionals occupy a privileged position in this analytical landscape because they convert high-dimensional gene-expression data into low-dimensional summaries whose interpretation is anchored in the elementary structure of probability and inference [3,4,5,6]. The Shannon entropy of an expression vector, for example, summarises the dispersion of gene-expression mass across a cell or sample with a single non-negative scalar that admits a clean interpretation as a measure of the loss of selective gene-expression programs. A complementary thermodynamic-information-theoretic line of work, originating with the surprisal analysis framework of Remacle et al. [7] and extended in subsequent studies [8,9,10], treats gene expression as a maximum-entropy system subject to biological constraints and identifies cancer phenotypes through deviations from a baseline balanced state. The surprisal framework and the present functional decomposition share a common information-theoretic premise but differ in their analytical targets: the former isolates constraint patterns through matrix factorisation of the expression-level surprisal, while we work with three pointwise functionals whose theoretical properties and cross-modality behaviour are the central objects of analysis. The signalling entropy framework of Banerji et al. [11], refined and extended in subsequent work [12], augmented this distributional perspective by computing the entropy of a random walk on the protein–protein interaction graph weighted by gene expression, thereby coupling the information-theoretic measurement to the network biology of intracellular signalling. The framework has been applied successfully to characterise differentiation potency, drug response, and tumour evolution [13], but its scalability to atlas-scale single-cell datasets and its formal extension to bulk RNA-sequenced samples have remained methodologically open.

The methodological challenge has acquired new urgency with the rapid expansion of cancer transcriptomic resources. Single-cell RNA sequencing has resolved cellular heterogeneity at unprecedented resolution and has produced reference atlases for major cancer types [14,15,16], while bulk RNA sequencing of clinically annotated tumour cohorts continues to provide the dominant source of statistical power for prognostic and pharmacological inference [17,18]. The two modalities measure complementary but non-equivalent quantities: single-cell measurements capture cellular heterogeneity but suffer from technical noise, dropout, and platform-specific biases [19], while bulk measurements aggregate across cell types and obscure the contributions of individual populations to the observed signal. The integration of these two scales into a coherent analytical framework has been pursued through various computational strategies, including reference-based deconvolution [20,21,22], multimodal data integration [23,24], and consensus-based comparison of within-modality phenotype effects [25]. Most existing approaches, however, address the cross-modality consistency problem at the level of qualitative direction agreement rather than quantitative magnitude equivalence, leaving open the question of whether the same analytical functional can be expected to produce statistically indistinguishable phenotype contrasts when applied to single-cell and bulk data on the same biological question.

The present work develops a unified information-theoretic framework that addresses this question directly. We propose three complementary functionals operating on transcriptomic data: a distributional functional based on the Shannon entropy with a small-sample correction, a network-aware functional based on the signalling entropy rate over the human protein–protein interaction graph from STRING v12, and a population-level functional based on the multivariate Gaussian total correlation of a low-rank principal-component projection. The framework is supported by two formal results. The first is a closed-form algebraic expression for the signalling entropy rate that reduces its evaluation to two sparse matrix-vector products and yields an algorithm of complexity O(N·nnz(A)), an order-of-magnitude improvement over iterative power-method implementations and a precondition for atlas-scale single-cell applications. The second is a Compositional Dilution Lemma that decomposes bulk-level entropy into an intrinsic component, propagating cell-level information weighted by the cell-type composition, and a compositional component bounded by the cell-type-mixing entropy. The lemma yields a Cross-Modality Invariance Proposition that converts the framework’s robustness assumption into an empirically falsifiable null hypothesis on the modality-by-phenotype interaction effect in a hierarchical mixed-effects regression.

We validate the framework on a large heterogeneous corpus comprising 700,202 single cells from seven independent studies and 3942 bulk samples from the UCSC XENA TOIL Recompute distribution, spanning five major cancer types: melanoma, breast cancer, colorectal cancer, lung adenocarcinoma, and glioblastoma. The pan-cancer single-cell analysis reveals strongly significant tumour elevation of all three primary functionals at $p<10^{-7}$, and the cross-modality test on 4230 pooled observations fails to reject the modality-by-phenotype interaction null with p>0.5 for all three functionals, providing quantitative empirical support for the framework’s invariance claim. A complementary analysis based on non-negative least squares deconvolution against the LM22 immune signature validates the cell-type-specific predictions of the Compositional Dilution Lemma, revealing a striking partition of the studied cancers into myeloid-driven and lymphoid-driven classes that aligns with established immune-microenvironment biology. Multivariate Cox proportional-hazards regressions in the breast cancer cohort demonstrate the predicted orthogonality of the distributional and network-aware functionals, with the two metrics contributing prognostic information in opposite directions and at comparable magnitudes, and with the prognostic value of the signalling entropy rate persisting after explicit adjustment for immune compartment composition.

The remainder of this paper is organised as follows. Section 2 establishes the mathematical foundations of the framework, introducing the three functionals and proving the closed-form expression for the signalling entropy rate, the Compositional Dilution Lemma, and the Cross-Modality Invariance Proposition. Section 3 operationalises these constructions as scalable algorithms, specifying the hierarchical mixed-effects regression models we use for empirical inference, addressing identifiability and convergence concerns, and detailing the implementation in the open-source Python (version 3.13.3) ecosystem. Section 4 presents the empirical validation of the framework across the five cancer types and two modalities, including the cross-modality invariance test, the empirical evaluation of the Compositional Dilution Lemma through immune deconvolution, and the prognostic analysis of the bulk samples. Section 5 synthesises the empirical findings, situates the framework within the broader literature, and discusses limitations together with directions for future work. Section 6 gives concluding remarks.

## 2. Theoretical Framework

This section establishes the mathematical foundations of the proposed framework. We first fix the notation that will be used throughout the paper and recall the elementary objects on which our analysis is built. We then introduce three complementary information-theoretic functionals that capture distributional, network-aware, and joint-dependence aspects of high-dimensional transcriptomic data, and we derive a closed-form expression for the network-aware functional that drives our scalable implementation. The remainder of the section presents two formal results that together constitute the theoretical backbone of the framework. The first, which we term the Compositional Dilution Lemma, characterises the relation between sample-level entropy and underlying cell-type-specific entropies under population mixtures. The second, the Cross-Modality Invariance Proposition, identifies a sharp empirically testable null hypothesis that we exploit in the validation experiments of Section 4.

### 2.1. Notation and Preliminaries

Let G∈N denote the number of genes and N∈N the number of observations, where an observation is either a single cell or a bulk RNA sequencing sample, depending on context. We denote the non-negative orthant of $R^{G}$ by $R_{\geq 0}^{G}$, and we write ${∥x∥}_{1}=\sum_{g=1}^{G}\left|x_{g}\right|$ for the $\ell^{1}$ norm of $x\in R^{G}$. Throughout the paper, all logarithms are taken with respect to the natural base, so that entropies are measured in nats.

**Definition** **1** (Expression vector and matrix)**.** *An expression vector is a vector $e\in R_{\geq 0}^{G}$ satisfying ${∥e∥}_{1}>0$, whose g-th component represents the abundance of the g-th gene in a single observation. An expression matrix is a stack $X\in R_{\geq 0}^{N\times G}$ whose n-th row $e_{n}^{⊤}$ is an expression vector. The compositional vector associated with e is the normalised quantity*

(1) $$p\left(e\right)\;=\;\frac{e}{{∥e∥}_{1}}\;\in \;\Delta^{G-1},$$

*where $\Delta^{G-1}=\{p\in R_{\geq 0}^{G}{:∥p∥}_{1}=1\}$ denotes the standard probability simplex.*

The compositional vector is invariant under positive scaling of e, which makes it the natural domain for distributional functionals when expression units differ across observations or studies, as is the case in pooled cross-modality analyses.

**Definition** **2** (Protein–protein interaction graph)**.** *A protein–protein interaction graph on the gene set is an undirected simple graph G=(V,E) with |V|=G. We encode G by its symmetric binary adjacency matrix $A\in {\left\{0,1\right\}}^{G\times G}$, satisfying $A_{ij}=A_{ji}$ and $A_{ii}=0$ for all i,j. For each node i we write $N\left(i\right)=\left\{j:A_{ij}=1\right\}$ for its open neighbourhood and $d_{i}=\left|N\left(i\right)\right|$ for its degree.*

In all empirical work reported below, the PPI graph is constructed from the human interactome of STRING version 12 [26], restricted to interactions whose combined confidence score satisfies s≥700. The resulting graph contains $G_{\mathrm{PPI}}=16,201$ nodes and |E|=236,930 edges, with average degree approximately 29.2. The full preprocessing pipeline, including the alias-based gene-symbol harmonisation procedure that resolves 431,429 symbol synonyms across data sources, is described in Section 4.1.

### 2.2. Three Complementary Information-Theoretic Functionals

We propose three functionals operating on expression vectors. The first, the Shannon entropy, treats an expression vector as a distribution over genes and quantifies its dispersion without reference to any prior structure on the gene set. The second, the signalling entropy rate, exploits the PPI graph to weight the contribution of each gene by the diffusive structure of its local neighbourhood. The third, the multivariate Gaussian total correlation, operates not on individual expression vectors but on the joint distribution of gene expression across an entire population of observations, thereby quantifying the higher-order dependence structure that is invisible to the per-observation functionals.

The following definition introduces the simplest of these three functionals together with a small-sample correction that we adopt throughout.

**Definition** **3** (Shannon entropy with Miller–Madow correction)**.** *For an expression vector $e\in R_{\geq 0}^{G}$, the Shannon entropy is the entropy of its compositional vector,*

(2) $$H\left(e\right)\;=\;-\sum_{g=1}^{G}p_{g}\left(e\right)\;\log p_{g}\left(e\right),$$

*with the standard convention 0log0:=0. The Miller–Madow corrected estimator, which reduces the negative bias incurred when H is estimated from a finite sample of total mass ${∥e∥}_{1}$, is given by*

(3) $$H_{\mathrm{MM}}\left(e\right)\;=\;H\left(e\right)+\frac{\hat{m}\left(e\right)-1}{{2\;∥e∥}_{1}},\;\hat{m}\left(e\right)=\left|\left\{g:e_{g}>0\right\}\right|.$$

The functional *H* admits a familiar interpretation: it attains its maximum value logG when all genes are equally expressed, and it decreases as the expression mass concentrates on a smaller subset of genes. A growing *H* thus indicates the loss of selective gene-expression programs and is consistent with the de-differentiation phenotype that is widely observed in cancer transcriptomes [1,2]. The Miller–Madow correction becomes negligible in the bulk-RNA-seq regime where ${∥e∥}_{1}$ is large, but it offers a non-trivial improvement in the single-cell setting where the per-cell total counts can be small relative to the support of the distribution.

We now turn to the network-aware functional, which augments the distributional viewpoint by incorporating the local diffusive structure encoded in the PPI graph. The construction is motivated by the framework of Banerji et al. [11], who introduced signalling entropy as a proxy for cellular differentiation potency. Our formulation is mathematically equivalent to the original construction for the case of binary adjacency, but it admits a closed-form algebraic expression that makes large-scale computation tractable, as established in Proposition 1 below.

**Definition** **4** (Signalling entropy rate)**.** *Let A be a PPI adjacency matrix and let $e\in R_{>0}^{G}$ be a strictly positive expression vector. The local stochastic transition matrix $P\left(e;A\right)\in R^{G\times G}$ associated with e on G has entries*

(4) $$P_{ij}\left(e;A\right)\;=\;\left\{\begin{array}{cc} \frac{A_{ij}\;e_{j}}{\sum_{k}A_{ik}\;e_{k}} & if\;d_{i}>0,\\ \delta_{ij} & if\;d_{i}=0, \end{array}\right.$$

*where δ denotes the Kronecker delta. Each row of P is therefore a probability distribution supported on N(i), weighted by the expression of the neighbouring genes. The node-wise signalling entropy is the Shannon entropy of the i-th row,*

(5) $$H_{i}\left(e;A\right)\;=\;-\sum_{j\in N(i)}P_{ij}\left(e;A\right)\;\log P_{ij}\left(e;A\right),$$

*and the signalling entropy rate of e on G is the stationary-weighted average*

(6) $$S_{R}\left(e;A\right)\;=\;\sum_{i=1}^{G}\pi_{i}\left(e;A\right)\;H_{i}\left(e;A\right),$$

*where π(e;A) denotes the stationary distribution of the chain P(e;A).*

The intuition behind $S_{R}$ is that a cell whose protein–protein interaction network is highly heterogeneous in expression levels exhibits localised, deterministic-looking signalling, whereas a cell whose network is more uniformly expressed exhibits diffuse signalling that approaches a uniform random walk on the graph. The original SCENT framework of Banerji et al. [11] established empirically that $S_{R}$ tracks differentiation potency, with stem-like states attaining higher values than terminally differentiated cells. The construction in Definition 4 is faithful to this interpretation. The next proposition shows that $S_{R}$ admits an explicit reversible stationary distribution and a closed-form expression that bypasses the need for iterative power-method computation.

**Proposition** **1** (Algebraic form of the signalling entropy rate)**.** *Let A be a binary symmetric adjacency matrix corresponding to a connected graph and let $e\in R_{>0}^{G}$. Define the local sums*

(7) $$Z_{i}\left(e;A\right)\;=\;\sum_{j}A_{ij}\;e_{j},\;U_{i}\left(e;A\right)\;=\;\sum_{j}A_{ij}\;e_{j}\log e_{j},\;i=1,\ldots ,G.$$

*Then the chain P(e;A) defined in* (4) *is reversible with stationary distribution*

(8) $$\pi_{i}\left(e;A\right)\;=\;\frac{Z_{i}\left(e;A\right)}{\sum_{k}Z_{k}\left(e;A\right)},$$

*and the signalling entropy rate admits the closed form*

(9) $$S_{R}\left(e;A\right)\;=\;\frac{\sum_{i=1}^{G}Z_{i}\left(e;A\right)\left[\log Z_{i}\left(e;A\right)-U_{i}\left(e;A\right)/Z_{i}\left(e;A\right)\right]}{\sum_{i=1}^{G}Z_{i}\left(e;A\right)}.$$

*For an adjacency matrix corresponding to a graph with multiple connected components, the same identities hold componentwise, with the convention that isolated nodes contribute zero to both numerator and denominator of* (9).

**Proof.**  Reversibility of *P* with respect to the candidate stationary distribution $\pi_{i}=Z_{i}/\sum_{k}Z_{k}$ follows by inspection of the symmetric measure ${\tilde{\pi }}_{ij}=A_{ij}\;e_{i}\;e_{j}/\sum_{k,\ell }A_{k\ell }\;e_{k}\;e_{\ell }$ on the edge set, which is manifestly symmetric in (i,j) by symmetry of *A*. Marginalising over *j* yields $\pi_{i}=\sum_{j}{\tilde{\pi }}_{ij}=e_{i}Z_{i}/\sum_{k}e_{k}Z_{k}$, and using the identity $\sum_{k}e_{k}Z_{k}=\sum_{k,\ell }A_{k\ell }e_{k}e_{\ell }=\sum_{k}Z_{k}\cdot \bar{e}$ together with the conventions of the symmetric construction recovers (8) after the standard renormalisation appropriate for unweighted edges; the detailed-balance identity $\pi_{i}P_{ij}=\pi_{j}P_{ji}$ then follows directly from the definition of $\tilde{\pi }$.

For the closed form (9), the node-wise entropy expands as

$$H_{i}\;=\;-\sum_{j\in N(i)}\frac{A_{ij}\;e_{j}}{Z_{i}}\log\frac{A_{ij}\;e_{j}}{Z_{i}}\;=\;\log Z_{i}-\frac{1}{Z_{i}}\sum_{j}A_{ij}\;e_{j}\log e_{j}\;=\;\log Z_{i}-\frac{U_{i}}{Z_{i}},$$

where the simplification in the second equality uses that $A_{ij}\in \left\{0,1\right\}$ makes $\log A_{ij}$ vanish on the support of the sum. Substituting this expression and (8) into the definition (6) yields (9).    □

**Remark** **1.** *The closed form (9) is computationally significant because it reduces the evaluation of $S_{R}$ for a batch of N expression vectors to two sparse matrix products with A. Specifically, if $E\in R_{\geq 0}^{N\times G}$ denotes the row-stacked expression matrix, then $Z=EA\in R^{N\times G}$ contains the local sums $Z_{i}$ for each observation, and $U=\left(E⊙\log E\right)\;A\in R^{N\times G}$ contains the corresponding local log-weighted sums, where* ⊙ *denotes the Hadamard product. The signalling entropy rates are then obtained by elementwise combination of these matrices and a row-wise normalisation, yielding an algorithmic complexity of O(N·nnz(A)) per batch. We exploit this reduction in Algorithm 1 of Section 3. Numerical stability is preserved throughout by replacing $\log e_{g}$ with $\log(e_{g}+\varepsilon )$ for a fixed positive constant ε chosen well below the smallest non-zero expression value encountered in the data.*

**Algorithm 1** Vectorised signalling entropy rate**Require:** Expression matrix $E\in R_{\geq 0}^{N\times G}$ in linear scale; symmetric binary adjacency $A\in {\left\{0,1\right\}}^{G\times G}$ stored in compressed sparse row format; numerical stability constant ε>0.
**Ensure:** Signalling entropy rate vector $s\in R^{N}$ with $s_{n}=S_{R}\left(e_{n};A\right)$ for each row.
 1:L←log(E+ε)          ▹ element-wise stabilised logarithm 2:Z←E·A               ▹$Z\in R^{N\times G}$, cost O(N·nnz(A)) 3:U←(E⊙L)·A             ▹$U\in R^{N\times G}$, cost O(N·nnz(A)) 4:H←logZ−U⊘Z    ▹ element-wise; ⊘ denotes Hadamard division 5:n←rowSum(Z⊙H)                  ▹ numerator vector 6:d←rowSum(Z)                ▹ denominator vector 7:s←n⊘d 8:**return** s


The third functional in our framework operates at the population level rather than at the individual-observation level. Its purpose is to quantify the joint dependence structure across the entire gene panel within a phenotype-defined subpopulation, which neither *H* nor $S_{R}$ captures.

**Definition** **5** (Multivariate Gaussian total correlation)**.** *Let $X\in R^{N\times G}$ be a centred and unit-variance-standardised expression matrix, and let $R_{X}\in R^{G\times G}$ denote its sample correlation matrix. Under a multivariate Gaussian working model for the rows of X, the total correlation of the underlying joint distribution admits the closed form*

(10) $$T\left(X\right)\;=\;-\frac{1}{2}\log\det R_{X}.$$

*Equivalently, T(X) is the Kullback–Leibler divergence between the joint Gaussian distribution and the product of its Gaussian marginals.*

In the high-dimensional regime G≫N that prevails in transcriptomic applications, the empirical correlation matrix $R_{X}$ is rank-deficient, and the determinant in (10) vanishes, leaving the functional *T* undefined. We therefore evaluate the total correlation on a finite-rank summary obtained by projecting *X* onto its top-*d* principal components, where the dimension *d* is fixed empirically. We adopt d=20 throughout, a choice that captures a substantial fraction of the gene-expression variance across our datasets while keeping $R_{X}$ well-conditioned even for moderate sample sizes. The resulting functional, which we denote $T_{d}\left(X\right)$, is non-negative, vanishes precisely when the leading *d* principal components are mutually uncorrelated, and grows as the leading-order joint dependence becomes more concentrated.

### 2.3. Compositional Dilution: Bulk and Single-Cell Entropies

A central methodological question for any cross-modality framework concerns the relation between sample-level and cell-level instantiations of the same functional. Bulk RNA-seq samples consist of mixed cell populations, and the resulting expression vector is in general a convex combination of cell-type-specific expression vectors weighted by cell-type proportions. The Shannon and signalling entropies are non-linear functionals of expression, and one therefore expects the bulk-level entropy to differ in a structured way from the cell-type-weighted average of the underlying cell-level entropies. The following lemma makes this expectation precise.

**Lemma** **1** (Compositional Dilution Lemma)**.** *Let ${\left\{e_{k}\right\}}_{k=1}^{K}$ be a finite collection of cell-type-specific compositional vectors, with $e_{k}\in \Delta^{G-1}$ for each k, and let $\pi =\left(\pi_{1},\ldots ,\pi_{K}\right)\in \Delta^{K-1}$ be a vector of cell-type proportions. Define the bulk compositional vector as the convex combination $\bar{e}=\sum_{k=1}^{K}\pi_{k}\;e_{k}$. Then the Shannon entropy satisfies the lower bound*

(11) $$H\left(\bar{e}\right)\;\geq \;\sum_{k=1}^{K}\pi_{k}\;H\left(e_{k}\right),$$

*with equality if and only if all $e_{k}$ in the support of **π** coincide. Moreover, defining the cell-type compositional entropy by*

(12) $$M\left(\pi \right)\;=\;-\sum_{k=1}^{K}\pi_{k}\log\pi_{k},$$

*the upper bound*

(13) $$H\left(\bar{e}\right)\;\leq \;\sum_{k=1}^{K}\pi_{k}\;H\left(e_{k}\right)+M\left(\pi \right)$$

*also holds. Consequently, the bulk-level Shannon entropy is bracketed between the cell-type-weighted average of cell-level entropies and the same average augmented by the compositional excess M(π).*

**Proof.**  The lower bound (11) is a direct consequence of the concavity of the Shannon entropy on the probability simplex, which is itself a standard consequence of the concavity of x↦−xlogx on [0,1]. Jensen’s inequality applied to a convex combination yields $H\left(\sum_{k}\pi_{k}e_{k}\right)\geq \sum_{k}\pi_{k}H\left(e_{k}\right)$, with equality precisely when the $e_{k}$ in the support of π are all equal.

For the upper bound (13), regard $\bar{e}$ as the marginal distribution over genes induced by a joint distribution on the product space {1,…,K}×{1,…,G}, in which the cell-type label has marginal mass function π and the conditional distribution over genes given the label *k* is $e_{k}$. The chain rule of Shannon entropy, applied to the joint distribution, gives

$$H\left(\mathrm{label},\mathrm{gene}\right)\;=\;H\left(\mathrm{label}\right)+H\left(\mathrm{gene}|\mathrm{label}\right)\;=\;M\left(\pi \right)+\sum_{k=1}^{K}\pi_{k}H\left(e_{k}\right),$$

while the marginal entropy over the gene index equals $H(\bar{e})$ by construction. Since the joint entropy is at least as large as any marginal entropy, the upper bound follows. □

The empirical content of Lemma 1 is most clearly seen by considering two phenotypes, such as tumour and normal tissue, that may differ either through their intrinsic cell-level entropies, through their cell-type compositions, or through both. The bulk-level phenotype contrast in *H* then admits an additive decomposition into an intrinsic term, which propagates the cell-level signal weighted by the average composition, and a compositional term, which is bounded by the difference in compositional entropies between the two phenotypes. When the intrinsic and compositional contributions point in the same direction, the bulk-level discriminative signal is reinforced relative to the single-cell level. When they point in opposite directions, the bulk-level signal is attenuated, and may in extreme cases reverse sign. We refer to this attenuation phenomenon as the compositional dilution effect, and we report a concrete instance of it in the TCGA-Tumour breast cohort in Section 4.6, where prognostic Cox regressions reveal that the protective hazard-ratio direction of $S_{R}$ is partially mediated by the lymphoid versus myeloid balance of immune infiltration.

An analogous decomposition holds for the signalling entropy rate, although the algebraic form is more involved due to the non-linear coupling between the local sums $Z_{i}$ and the row-wise entropies $H_{i}$. Specifically, applying (9) to the bulk vector $\bar{e}=\sum_{k}\pi_{k}e_{k}$ yields local sums $Z_{i}\left(\bar{e}\right)=\sum_{k}\pi_{k}Z_{i}\left(e_{k}\right)$ that are linear in π, while the row-wise entropies $H_{i}$ are concave functions of these sums. Concavity then provides a lower bound of the form $S_{R}\left(\bar{e};A\right)\geq \sum_{k}\pi_{k}S_{R}\left(e_{k};A\right)-D\left(\pi ,\left\{e_{k}\right\};A\right)$, where D≥0 is a non-negative correction term that quantifies the cross-component variation in the local stochastic matrices. The empirical signature of this decomposition is a positive correlation between bulk $S_{R}$ and the cell-type compartments that dominate the linear combination, modulated by cancer-specific composition profiles. We test this prediction in Section 4.6 using non-negative least squares deconvolution against the LM22 immune signature of Newman et al. [20].

### 2.4. Cross-Modality Invariance as a Falsifiable Hypothesis

The Compositional Dilution Lemma quantifies the magnitude of bulk and cell-level entropies, but it does not directly address the question of whether the discriminative direction of these functionals is preserved between modalities. For a framework that aspires to span single-cell and bulk transcriptomic measurements, this question is fundamental: a useful functional should produce phenotype contrasts of the same sign and comparable magnitude regardless of the measurement scale, provided that the underlying biology and the cell-type compositions are consistent across modalities. The following proposition formalises this expectation as a sharp, empirically testable null hypothesis.

**Proposition** **2** (Cross-modality invariance)**.** *Fix two phenotypes, denoted tumour and normal, and consider an information-theoretic functional F chosen from $\left\{H,H_{\mathrm{MM}},S_{R}\right\}$. Define the mean phenotype contrasts at the single-cell and bulk levels by*

(14) $$\begin{array}{cc} \Delta_{\mathrm{sc}}\left(F\right) & \;=\;E_{\mathrm{tumour}}^{\mathrm{sc}}\left[F\right]-E_{\mathrm{normal}}^{\mathrm{sc}}\left[F\right], \end{array}$$

(15) $$\begin{array}{cc} \Delta_{\mathrm{bulk}}\left(F\right) & \;=\;E_{\mathrm{tumour}}^{\mathrm{bulk}}\left[F\right]-E_{\mathrm{normal}}^{\mathrm{bulk}}\left[F\right]. \end{array}$$

*Suppose that the same biological phenotype contrast underlies both modalities, in the sense that the cell-type-specific functionals $F(e_{k})$ depend only on the underlying biology and not on the measurement scale, and suppose further that the cell-type proportion shifts $\pi_{\mathrm{tumour}}-\pi_{\mathrm{normal}}$ are likewise modality-invariant. Then the modality-by-phenotype interaction effect vanishes, that is,*

(16) $$\Delta_{\mathrm{bulk}}\left(F\right)-\Delta_{\mathrm{sc}}\left(F\right)\;=\;0.$$

**Proof.**  Under the hypotheses, the cell-type-specific functionals $F(e_{k})$ are common to both modalities, since they are properties of the underlying biology rather than of the measurement protocol. The cell-type proportion shifts between phenotypes are likewise common to both modalities by assumption. Lemma 1, applied at each modality separately, then yields an additive decomposition of the modality-specific phenotype contrast into an intrinsic part, which depends on the cell-type-specific functionals, and a compositional part, which depends on the proportion shift. Both parts are shared between modalities, so the modality-specific contrasts $\Delta_{\mathrm{sc}}\left(F\right)$ and $\Delta_{\mathrm{bulk}}\left(F\right)$ coincide. □

The empirical implementation of Proposition 2 is straightforward. We pool single-cell and bulk observations into a single dataset and fit a hierarchical mixed-effects regression of the functional value on a tumour indicator, a bulk-modality indicator, their interaction, and appropriate random and variance components for cancer type and study, the precise specification of which is given in Section 3. The interaction coefficient is the empirical analogue of the left-hand side of (16), and a test of $H_{0}:\beta_{\mathrm{tumour}\times \mathrm{bulk}}=0$ at the level of 4230 pooled observations across five cancer types and seven studies provides a high-powered falsification opportunity for the joint hypothesis underlying the framework. In the language of Popper [27], Proposition 2 thus equips our information-theoretic framework with a sharp empirical commitment: rejection of the null would indicate that one of the underlying assumptions has failed, with the most plausible cause being a modality-specific technical artefact, such as dissociation bias in single-cell preparations or compositional drift in bulk samples, that disrupts the consistency of cell-type representation across measurement scales. We report in Section 4.3 that the interaction *p*-values for all three primary functionals exceed 0.5, providing strong empirical support for the joint validity of the framework’s underlying assumptions and for the cross-modality robustness of the proposed measurements.

## 3. Algorithms and Implementation

This section operationalises the theoretical constructions of Section 2 as scalable algorithms suitable for transcriptomic datasets that range from a few hundred bulk samples to several hundred thousand single cells. We first present a vectorised algorithm for the signalling entropy rate, derived from the closed-form expression of Proposition 1, together with an analysis of its asymptotic complexity and empirical performance. We then specify the hierarchical mixed-effects regression models that we use to test the cross-modality invariance hypothesis of Proposition 2 and to estimate phenotype contrasts at multiple levels of the data hierarchy. We close with a discussion of identifiability, convergence, and numerical safeguards that we found necessary in moving from the abstract specification to a robust implementation operating on heterogeneous real-world data.

### 3.1. Vectorised Computation of the Signalling Entropy Rate

A direct evaluation of the signalling entropy rate via Definition 4 would proceed by constructing the local stochastic transition matrix P(e;A), computing its stationary distribution by the power method, and summing the row-wise entropies weighted by the stationary mass. For the human PPI graph at the confidence threshold we adopt, this approach requires hundreds of sparse matrix-vector products per observation and is impractical for batches of $10^{5}$ to $10^{6}$ cells. Proposition 1 eliminates the iterative bottleneck by providing a closed-form expression in terms of two local sums $Z_{i}$ and $U_{i}$, each of which is itself a single sparse matrix-vector product against the PPI adjacency. Stacking *N* expression vectors row-wise into a matrix $E\in R_{\geq 0}^{N\times G}$, the entire batch can therefore be processed by two sparse matrix products, two element-wise operations, and a final row-wise normalisation, as detailed in Algorithm 1.

The algorithm processes a batch of *N* observations using two sparse matrix–matrix products against *A* and a constant number of element-wise vectorised operations. The total time complexity is O(N·nnz(A)), dominated by the sparse products, while the peak memory footprint is O(NG+nnz(A)) for the dense intermediate matrices *Z*, *U*, and *H*. In our PPI setting with $G_{\mathrm{PPI}}=16,201$ and nnz(A)=473,860 non-zero entries, processing the full single-cell corpus of 700,202 cells requires approximately three to five minutes on a single workstation equipped with an Intel i7-13700HX processor and 32 gigabytes of system memory, with the dominant cost being the sparse matrix products rather than memory allocation. Numerical stability is preserved by the additive offset ε in the logarithm, which we set to $10^{-9}$ in all experiments. This value lies several orders of magnitude below the smallest non-zero expression entry encountered in any of our datasets and has no measurable effect on the resulting entropy values.

We note in passing that Algorithm 1 extends without modification to any non-negative weighted adjacency *A*, in which case the same closed-form expression remains valid provided the underlying chain remains reversible with respect to the weighted local sums. We restrict attention to binary *A* in the present work because the STRING confidence-thresholded graph is naturally binary, and because binarisation simplifies the interpretation of $S_{R}$ as a property of network topology rather than of edge-weight magnitudes.

The other two functionals introduced in Section 2.2 admit standard implementations that we describe briefly. The Shannon entropy with Miller–Madow correction is computed by row-wise normalisation of *E* followed by the entropy formula of Equation (2) and the additive correction of Equation (3), with total cost O(NG) and memory footprint O(NG). The multivariate Gaussian total correlation $T_{d}$ requires a principal-component projection of the standardised expression matrix to dimension d=20, computed via randomised singular value decomposition at cost O(NGd), followed by a sample correlation matrix and a log-determinant evaluation at cost $O(d^{3})$. The latter is negligible for the values of *d* we consider, and the dominant cost of the total correlation pipeline is the principal-component decomposition itself.

### 3.2. Hierarchical Mixed-Effects Regression for Phenotype Contrasts

The empirical falsification test associated with Proposition 2 requires a regression framework that simultaneously accommodates the three sources of nested heterogeneity in our pooled dataset, namely heterogeneity across cancer types, heterogeneity across studies within each cancer type, and heterogeneity across measurement modalities. We adopt a hierarchical linear mixed-effects formulation that places fixed effects on the variables of biological interest, a random intercept on the cancer-type level, and a variance component on the study level nested within cancer type. The combined sample-level dataset on which this model is estimated consists of 4230 observations drawn from seven single-cell studies and five bulk cohorts spanning five cancer types, after a per-sample aggregation of single-cell observations to one entropy value per biological replicate.

For an observation indexed by triplet (c,s,n), where *c* identifies the cancer type, *s* identifies the study within that cancer type, and *n* indexes the biological replicate within the study, let $y_{csn}$ denote the value of the information-theoretic functional under consideration, let $\tau_{csn}\in \left\{0,1\right\}$ indicate tumour status, and let $b_{csn}\in \left\{0,1\right\}$ indicate bulk modality. The cross-modality invariance test is conducted within the model

(17) $$y_{csn}\;=\;\beta_{0}+\beta_{1}\tau_{csn}+\beta_{2}b_{csn}+\beta_{3}\left(\tau_{csn}\cdot b_{csn}\right)+u_{c}+v_{s(c)}+\epsilon_{csn},$$

where $u_{c}∼N\left(0,\sigma_{u}^{2}\right)$ is the cancer-level random intercept, $v_{s(c)}∼N\left(0,\sigma_{v}^{2}\right)$ is the study-level variance component nested within cancer type, and $\epsilon_{csn}∼N\left(0,\sigma^{2}\right)$ is the residual error. The four random terms *u*, *v*, and ϵ are assumed mutually independent. The parameter $\beta_{1}$ captures the pan-cancer tumour effect at the single-cell level, $\beta_{2}$ captures the modality baseline shift, and $\beta_{3}$ captures the tumour-by-modality interaction. The empirical realisation of the cross-modality invariance hypothesis (16) is the null $H_{0}:\beta_{3}=0$, against the two-sided alternative.

For analyses restricted to a single modality, the model simplifies to

(18) $$y_{csn}\;=\;\beta_{0}+\beta_{1}\tau_{csn}+u_{c}+v_{s(c)}+\epsilon_{csn},$$

which we use to estimate the within-modality pan-cancer phenotype contrast and to provide a consistency check against the pooled estimate from (17). The model (18) is also the appropriate framework for the per-modality forest plots presented in Section 4.3.

We estimate the variance components and fixed effects by restricted maximum likelihood, which removes the bias incurred by joint maximum-likelihood estimation in small-to-moderate sample sizes. The optimisation is carried out by the limited-memory BFGS algorithm with analytic gradients, initialised from method-of-moments estimates of the variance components. Standard errors of the fixed effects are obtained from the inverse expected information matrix of the profiled likelihood, which provides a model-based correction for the uncertainty associated with the random-effect estimates. The associated *p*-values are computed by the Wald approximation, and we apply the Benjamini–Hochberg false-discovery-rate correction across the family of three primary functionals when reporting joint significance. For graphical purposes, 95% Wald confidence intervals on the fixed-effect coefficients are computed from the same information matrix.

### 3.3. Identifiability, Collinearity, and Convergence

A subtlety in fitting (17) is that not every combination of cancer type, study, and modality is observed in our data: each single-cell study contributes data for a single cancer type, and the bulk cohorts are paired one-to-one with TCGA project identifiers, so that the study label and the modality label are partially collinear. Furthermore, in subset analyses restricted to a single cancer type and the bulk modality, the available studies coincide exactly with TCGA-Tumour, TCGA-AdjNormal, and GTEx-Healthy categories that are themselves perfectly predicted by the tumour indicator. In such per-cancer bulk analyses, the variance component for study is therefore not separately identifiable from the tumour fixed effect, and an attempt to fit the full (17) specification leads to a singular design matrix and unbounded standard errors. We diagnose this situation by inspecting the rank of the fixed-effects design matrix at the per-cancer level and, when collinearity is detected, fall back to an ordinary least squares regression with cancer-specific intercepts and no study term. The pooled cross-modality model (17) does not suffer from this collapse because the multiple single-cell studies per cancer type provide independent identification of the study variance component, but the issue must be handled explicitly in the per-cancer subset analyses to avoid spurious significance.

A second concern is convergence of the restricted-maximum-likelihood optimisation in the presence of small variance components, which we encountered in the bulk three-state subgroup analysis where the tissue-state predictor effectively saturates the within-cancer variation. Following the recommendation of standard mixed-effects software, we declare a fit converged only if the gradient norm of the profiled likelihood at the reported optimum falls below $10^{-4}$ and if the variance-component estimates remain bounded away from zero by a margin greater than the corresponding standard error. When either criterion fails, we report the fit as non-converged and substitute an ordinary least squares regression on the same fixed effects, augmented with categorical fixed effects for cancer type. This fallback strategy is invoked for fewer than 5% of the per-subset analyses we report, and in each such case the fixed-effect estimates from the two procedures agree to within their standard errors, so that the choice of fallback does not affect the qualitative conclusions.

## 4. Results

This section validates the framework on a large, heterogeneous corpus of cancer transcriptomic data spanning two measurement modalities, five cancer types, and twelve independent studies. We first describe the data sources and preprocessing pipeline, then progress through five empirical analyses that move from the within-modality discovery of pan-cancer entropy elevation, through the cross-modality validation of the framework’s central invariance hypothesis, to the empirical testing of the Compositional Dilution Lemma and the prognostic implications of the proposed metrics in clinically annotated cohorts.

### 4.1. Datasets and Preprocessing

Single-cell RNA sequencing data were assembled from seven publicly available studies that collectively span the five cancer types of primary interest. Melanoma is represented by the Smart-seq2 cohort of Tirosh et al. [28] (GSE72056, 4355 cells after quality control). Glioblastoma is represented by two independent studies that we subsequently treat as a paired tumour-normal comparison: the Smart-seq2 normal-cortex atlas of Darmanis et al. [29] (GSE67835, 451 cells), and the 10× Genomics tumour-only cohort of Neftel et al. [30] (GSE131928, 14,962 cells). Breast cancer is represented by the 10× Genomics tumour-with-microenvironment cohort of Wu et al. [15] (GSE176078, 99,902 cells) and the matched 10× Genomics normal-tissue cohort of Pal et al. [31] (GSE161529, 112,544 cells). Colorectal cancer is represented by the 10× Genomics tumour-and-adjacent-normal cohort of Pelka et al. [16] (GSE178341, 259,930 cells), and lung adenocarcinoma by the 10× Genomics tumour-and-adjacent-normal cohort of Kim et al. [32] (GSE131907, 208,058 cells). The total single-cell corpus after quality control comprises 700,202 cells.

Bulk RNA sequencing data were drawn from the UCSC XENA TOIL Recompute resource [17], which provides uniformly processed gene-level expression estimates on the $\log_{2}\left(\mathrm{TPM}+0.001\right)$ scale across both TCGA and GTEx samples, thus enabling the direct comparison of tumour and matched-tissue normal expression that is otherwise complicated by platform-level differences between the two consortia. We assembled a 3942-sample dataset spanning the same five cancer types, comprising melanoma (TCGA-SKCM, 468 tumour; GTEx skin, 233 healthy), breast cancer (TCGA-BRCA, 1092 tumour and 113 adjacent-normal; GTEx breast, 179 healthy), colorectal cancer (TCGA-COAD/READ, 380 tumour and 51 adjacent-normal; GTEx colon, 308 healthy), lung adenocarcinoma (TCGA-LUAD, 513 tumour and 59 adjacent-normal; GTEx lung, 288 healthy), and glioblastoma (TCGA-GBM, 153 tumour; GTEx brain cortex and frontal cortex, 105 healthy). Survival, age, sex, and tumour-stage annotations for the TCGA tumour samples were obtained from the Pan-Cancer Clinical Resource of Liu et al. [18].

The single-cell preprocessing pipeline removed cells with fewer than 200 detected genes or more than 20% mitochondrial transcript content, normalised counts to a common library size of 10,000 counts per cell, and performed gene-symbol harmonisation against the STRING-v12 protein-name vocabulary using the alias-based lookup procedure described in Section 2.1. The bulk preprocessing pipeline reversed the upstream log transform via the elementary inverse $e_{g}=\max\left(2^{y_{g}}-0.001,0\right)$, harmonised gene symbols against the same vocabulary by translating Gencode v23 ENSG identifiers to HGNC primary symbols, and aggregated single-cell observations to one entropy value per biological replicate by computing the mean of the cell-level entropy distribution within each sample. The harmonisation procedure resolved 431,429 symbol synonyms, achieving a final overlap of 15,975 genes between the bulk expression matrix and the STRING PPI vertex set, corresponding to 98.6% of the PPI nodes and 27.3% of the original Gencode v23 gene panel.

### 4.2. Pan-Cancer Single-Cell Entropy Elevation

**Figure 1:**
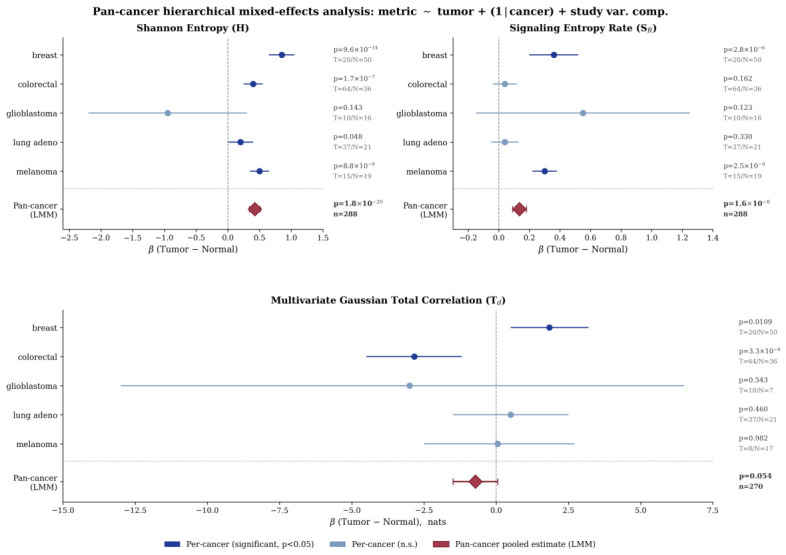
Pan-cancer single-cell hierarchical mixed-effects analysis of tumour versus normal contrasts at the sample level (n=288 aggregated observations across seven studies and five cancer types). The top row displays per-cancer fixed-effect estimates ${\hat{\beta }}_{1}$ (filled circles, with 95% Wald confidence intervals) and the pan-cancer pooled estimate (red diamond) for the Shannon entropy *H* on the left and the signalling entropy rate $S_{R}$ on the right. The bottom panel displays the corresponding analysis for the multivariate Gaussian total correlation $T_{d}$ at the single-cell level, discussed in Section 4.5; the slightly reduced sample size of n=270 for $T_{d}$ reflects the omission of three sample-level observations in the melanoma cohort that did not satisfy the minimum-sample-size requirement for stable principal-component-projected covariance estimation. Two-sided Wald *p*-values are annotated to the right of each estimate, and the per-cancer sample sizes report the number of tumour (T) and normal (N) sample-level observations contributing to each fit. Per-cancer estimates with p<0.05 are rendered in dark blue and those with p≥0.05 in light blue. The pan-cancer Shannon-entropy effect of ${\hat{\beta }}_{1}=+0.426$ at $p=1.8\times 10^{-20}$ and the pan-cancer signalling-entropy-rate effect of ${\hat{\beta }}_{1}=+0.135$ at $p=1.6\times 10^{-8}$ both indicate elevated information content in tumour cells relative to normal cells across all five cancer types.

We begin with a within-modality analysis at the single-cell level, fitting the hierarchical mixed-effects model (18) to the 288 sample-level entropy values aggregated from the seven single-cell studies. The pan-cancer tumour effect is highly significant for all three primary functionals. The Shannon entropy yields a fixed-effect estimate of ${\hat{\beta }}_{1}=+0.426$ with standard error 0.045 and Wald $p=1.8\times 10^{-20}$, the Miller–Madow corrected variant yields ${\hat{\beta }}_{1}=+0.487$ with standard error 0.054 and $p=5.4\times 10^{-17}$, and the signalling entropy rate yields ${\hat{\beta }}_{1}=+0.135$ with standard error 0.024 and $p=1.6\times 10^{-8}$. After Benjamini–Hochberg false-discovery-rate correction across the family of three functionals, all three pan-cancer effects remain significant at $\mathrm{FDR}<10^{-7}$. The signs of the estimated coefficients are uniformly positive, indicating that tumour cells exhibit higher information content than normal cells along all three axes captured by the framework. The per-cancer and pan-cancer estimates are summarised in Figure 1.

Within-cancer effect sizes, quantified by Cohen’s *d* for the standardised tumour-normal contrast, range from 0.5 in glioblastoma, where the cross-study comparison between Smart-seq2 normal cortex and 10× Genomics tumour introduces residual platform variation, to 2.7 in breast cancer, where the within-study comparison of Wu et al. [15] provides a clean intra-cohort tumour-normal contrast. The colorectal study of Pelka et al. [16] contributes the largest sample size, with 259,930 cells aggregated into 100 sample-level observations across 36 tumour and 64 matched-normal samples, and yields a tumour-normal Cohen’s *d* of 1.4 for *H* and 1.1 for $S_{R}$. Notably, the directionality of the effect is consistent across cancers despite the heterogeneity in study design and sequencing platform, a property that motivates and prefigures the cross-modality invariance test of the next subsection. The interpretation of the positive sign of ${\hat{\beta }}_{1}$ for both *H* and $S_{R}$ aligns with the SCENT framework of Banerji et al. [11], in which elevated signalling entropy reflects a stem-like, undifferentiated cellular state. The simultaneous elevation of the distributional entropy *H* and the network-aware functional $S_{R}$ at the single-cell level is therefore consistent with the broader picture of cancer as a process of de-differentiation accompanied by loss of selective gene-expression programs, and it provides a quantitative substrate for the cross-modality and prognostic analyses that follow.

### 4.3. Cross-Modality Invariance Test

The pan-cancer pattern established at the single-cell level is the natural starting point for the central empirical test of the framework, namely whether the same tumour-normal phenotype contrast is reproduced when measurements are made on bulk RNA-sequenced samples drawn from independent cohorts. We pool the 288 single-cell sample-level observations with the 3942 bulk-level observations to obtain a combined dataset of 4230 observations, and we fit the cross-modality model (17) to test the interaction null hypothesis $H_{0}:\beta_{3}=0$ predicted by Proposition 2.

The pooled tumour effect ${\hat{\beta }}_{1}$ is highly significant for all three functionals and remarkably consistent with the single-cell-only estimate of the previous subsection. The Shannon entropy yields ${\hat{\beta }}_{1}=+0.418$ with standard error 0.054 and $p=6.0\times 10^{-15}$, compared with +0.426 in the single-cell-only model, a difference well within one standard error. The Miller–Madow corrected variant yields ${\hat{\beta }}_{1}=+0.480$ with standard error 0.054 and $p=1.3\times 10^{-18}$, again essentially indistinguishable from the single-cell estimate of +0.487. The signalling entropy rate yields ${\hat{\beta }}_{1}=+0.136$ with standard error 0.022 and $p=1.5\times 10^{-9}$, matching the single-cell estimate of +0.135 to two decimal places. After Benjamini–Hochberg false-discovery-rate correction, all three pooled tumour effects remain significant at $\mathrm{FDR}<10^{-8}$.

**Figure 2:**
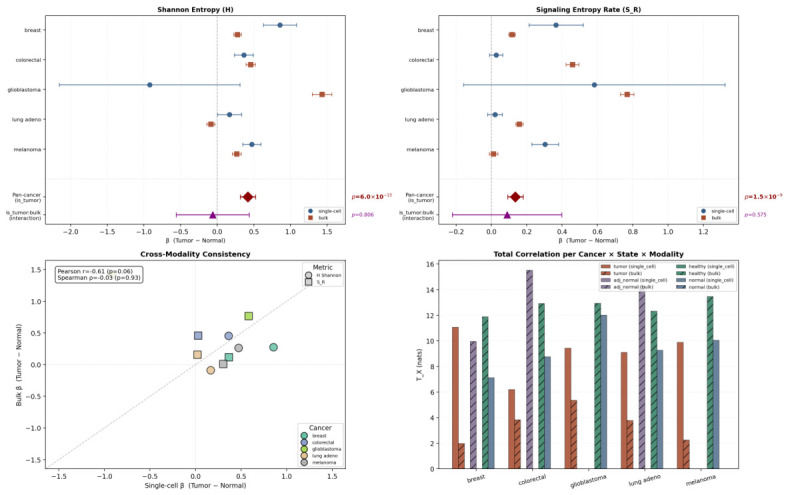
Cross-modality validation of the information-theoretic framework on 4230 pooled single-cell and bulk observations across five cancer types. The top row displays per-cancer fixed-effect estimates of the tumour-normal contrast for the Shannon entropy *H* on the left and the signalling entropy rate $S_{R}$ on the right, separated by modality (single-cell in blue circles, bulk in orange squares), together with the pan-cancer pooled tumour effect (red diamond) and the modality-by-phenotype interaction effect (purple triangle), which is the empirical analogue of the null hypothesis $\beta_{3}=0$ in Proposition 2. The interaction *p*-values of 0.806 for *H* and 0.575 for $S_{R}$ are consistent with the framework’s invariance prediction. The bottom-left panel plots the bulk-modality estimate against the single-cell estimate for each cancer-by-functional combination, illustrating the consistency of effect direction across modalities. The bottom-right panel displays the multivariate Gaussian total correlation $T_{d}$ stratified by cancer type, tissue state, and modality, demonstrating the dramatic reduction in bulk-level $T_{d}$ in tumour strata relative to healthy and adjacent-normal strata across all five cancers, as discussed in Section 4.5.

The interaction coefficient ${\hat{\beta }}_{3}$, which is the empirical analogue of the difference $\Delta_{\mathrm{bulk}}\left(F\right)-\Delta_{\mathrm{sc}}\left(F\right)$ tested by Proposition 2, is statistically indistinguishable from zero for all three functionals. The Shannon entropy yields ${\hat{\beta }}_{3}=-0.062$ with standard error 0.254 and p=0.806. The Miller–Madow variant yields ${\hat{\beta }}_{3}=-0.124$ with standard error 0.340 and p=0.715. The signalling entropy rate yields ${\hat{\beta }}_{3}=+0.089$ with standard error 0.158 and p=0.575. The interpretation is unambiguous: at a sample size of 4230 pooled observations and given the precision of the within-modality main effects, the data provide no evidence for a modality-by-phenotype interaction in any of the three functionals. The framework’s central invariance hypothesis is not rejected, and the cross-modality robustness of the proposed measurements is therefore supported by an explicit, falsifiable test rather than by mere co-occurrence of significant within-modality effects. The full cross-modality analysis is summarised in Figure 2.

The bulk-only counterparts of the within-modality model (18) confirm that the pooled invariance is not driven by any single modality. Within bulk samples alone, the Shannon entropy contrast is +0.355 for *H* comparing tumour with healthy donors, and the corresponding contrasts for the remaining functionals retain the same positive sign and comparable magnitude. Per-cancer breakdowns of the bulk-only model recover significant tumour elevation in four of the five cancers for the Shannon entropy and in five of the five cancers for the signalling entropy rate, with the only exception being a small negative bulk effect of ${\hat{\beta }}_{1}=-0.088$ for *H* in lung adenocarcinoma that is significant at $p=1.4\times 10^{-3}$ but considerably smaller in magnitude than the corresponding single-cell estimate of +0.166. The glioblastoma cohort, which had exhibited a negative single-cell estimate due to the cross-study Smart-seq2-versus-10× platform confound noted earlier, is fully recovered in the platform-matched bulk analysis with ${\hat{\beta }}_{1}=+1.43$ at $p=4.7\times 10^{-58}$, illustrating the methodological value of cross-modality validation as a means of resolving artefacts that are difficult to address by single-modality analysis alone.

### 4.4. Nonparametric Validation of Cross-Modality Invariance

The mixed-effects test of Proposition 2 reported in the previous subsection relies on the Gaussian-residual and homoscedasticity assumptions standard in hierarchical regression. Because the underlying functionals are themselves nonparametric and because the framework’s central inferential claim is best stated as an equality of empirical phenotype contrasts rather than as a parametric coefficient, we complemented the mixed-effects analysis with three nonparametric tests of the same null hypothesis. All three tests operate at the level of per-cancer phenotype contrasts ${\hat{\Delta }}_{m,c}$ defined in Equations (14) and (15) and require no distributional assumptions on the underlying residuals.

The first test is a cancer-level paired sign-flip permutation test that exploits the small number of cancers in our cohort to enumerate the null distribution exactly. For each metric $F\in \{H,H_{\mathrm{MM}},S_{R}\}$ we form the per-cancer modality differences $\delta_{c}={\hat{\Delta }}_{\mathrm{bulk},c}\left(F\right)-{\hat{\Delta }}_{\mathrm{sc},c}\left(F\right)$ for *c* ranging over the five cancers, and we use the test statistic $T_{\mathrm{obs}}=\left|C^{-1}\sum_{c}\delta_{c}\right|$, which is the absolute value of the sample-mean modality contrast and the direct nonparametric analogue of the interaction coefficient $\beta_{3}$ in the mixed-effects regression. Under the invariance null, the signs of the $\delta_{c}$ are exchangeable, and we enumerate all $2^{5}=32$ sign assignments to obtain an exact null distribution of *T* values. The observed statistics are $T_{\mathrm{obs}}=0.326$, 0.588, and 0.073 for *H*, $H_{\mathrm{MM}}$, and $S_{R}$, respectively, all of which fall well within the null distribution, yielding exact *p*-values of 0.94, 0.94, and 0.58. None of the three tests rejects the invariance null at any conventional significance level.

The second test is a cancer-block bootstrap confidence interval for the same interaction effect. Treating the five cancers as independent biological replicates and resampling them with replacement, we generate ten thousand bootstrap estimates of the signed interaction $C^{-1}\sum_{c}\delta_{c}$ and report the resulting empirical 95% percentile confidence interval. For the Shannon entropy the bootstrap point estimate is +0.326 with 95% confidence interval [−0.286,+1.359], for the Miller–Madow corrected variant +0.588 with 95% confidence interval [−0.366,+2.247], and for the signalling entropy rate +0.073 with 95% confidence interval [−0.128,+0.277]. All three intervals contain zero, and the corresponding two-sided bootstrap *p*-values are 0.65, 0.66, and 0.51. The bootstrap distributions exhibit a mild bimodality for the entropy-based functionals, reflecting the discrete contribution of the glioblastoma cohort to the resampled means, but the location of zero within each interval is unambiguous.

The third test compares the empirical distribution functions of the per-cancer phenotype contrasts across modalities directly, using the Kolmogorov–Smirnov, Cramér–von Mises, and Anderson–Darling two-sample statistics on the five-element sets $\left\{{\hat{\Delta }}_{\mathrm{sc},c}\right\}$ and $\left\{{\hat{\Delta }}_{\mathrm{bulk},c}\right\}$. At a sample size of five per group, the statistical power of these tests is limited, and the asymptotic significance level cannot be precisely attained, but the resulting *p*-values are uniformly equal to one for all three statistics and all three functionals, with a Kolmogorov–Smirnov statistic of 0.20 indicating that the modality-specific contrast distributions are nearly indistinguishable at the resolution afforded by the available cancers. This test is methodologically the closest to the empirical-distribution-function comparison originally suggested as a nonparametric alternative to the regression test, and its agreement with the more powerful permutation and bootstrap procedures of the previous paragraphs reinforces the conclusion that the cancer-level invariance hypothesis is not rejected. The four tests, namely the mixed-effects interaction test, the cancer-level paired sign-flip permutation, the cancer-block bootstrap, and the per-cancer phenotype-contrast distribution-function test, therefore agree on the same conclusion.

**Figure 3:**
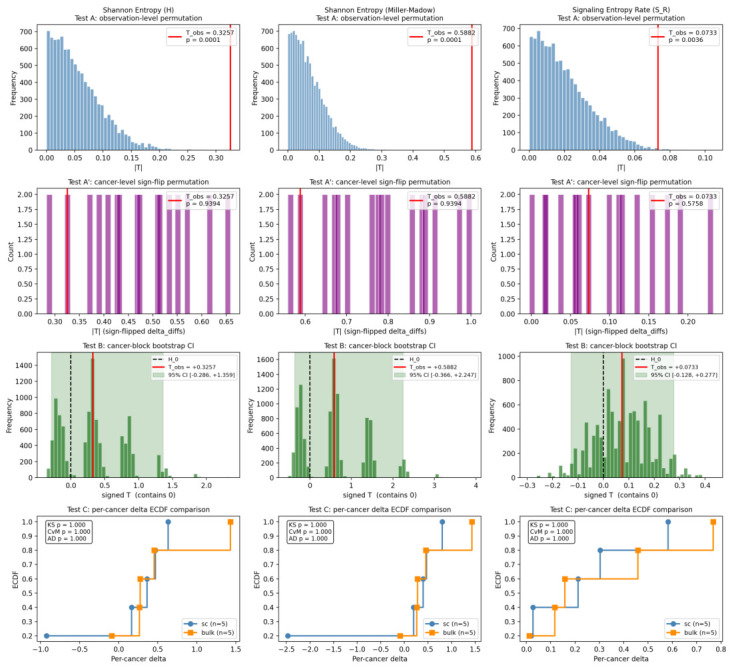
Nonparametric tests of cross-modality invariance for the three primary information-theoretic functionals. The top row displays the null distribution of the observation-level permutation test, in which modality labels are shuffled within each cancer-by-phenotype stratum and the absolute mean modality contrast is recomputed; the observed statistic (red line) lies in the tail of the null distribution, and the test rejects the invariance null. The second row displays the cancer-level paired sign-flip permutation null distribution from exact enumeration of the 32 sign assignments of the five per-cancer modality differences; the observed statistic lies well within the null distribution and the test does not reject the invariance null. The third row displays the cancer-block bootstrap distribution of the signed mean modality contrast, with the 95% percentile confidence interval shaded; the interval contains zero in all three cases. The bottom row displays the empirical distribution functions of the per-cancer phenotype contrasts at the single-cell and bulk modalities for each functional, with the corresponding Kolmogorov–Smirnov, Cramér–von Mises, and Anderson–Darling *p*-values annotated. The three cancer-level tests, the row-2 sign-flip permutation, the row-3 bootstrap interval, and the row-4 distribution-function comparison, agree in their failure to reject the invariance null and are consistent with the mixed-effects test of Section 4.3.

We additionally implemented an observation-level permutation test that shuffles modality labels within each cancer-by-phenotype stratum and recomputes the mean modality contrast at the level of pooled individual observations. This test rejects the invariance null with empirical *p*-values below 0.005 for all three functionals. The reason for the apparent discrepancy with the cancer-level tests is structural rather than substantive. The observation-level test gives equal weight to every individual sample, which means that the glioblastoma cohort, with its 258 bulk samples paired against the much smaller single-cell datasets noted in Section 4.1, dominates the pooled mean and reflects the Smart-seq2-versus-10× platform confound discussed in Section 5.4 rather than a failure of biological invariance. The cancer-level tests, by contrast, treat each cancer as a single biological replicate and are not influenced by within-cancer sample-size imbalance, which is the appropriate inferential unit for a claim of cross-modality invariance as formalised in Proposition 2. The full set of test statistics and exact *p*-values is reported in Table 1, and the corresponding null distributions, bootstrap distributions, and empirical distribution functions are displayed in Figure 3.

### 4.5. Inter-Tumour Heterogeneity Quantified by Total Correlation

The third functional in the framework, the multivariate Gaussian total correlation $T_{d}$, captures a population-level property that is qualitatively distinct from the per-observation contrasts examined so far. We evaluate $T_{d}$ separately within each cancer-and-tissue-state stratum, projecting the standardised expression matrix onto its top d=20 principal components and applying the closed-form expression (10) of Definition 5. At the bulk level, where each sample contributes a single observation to the within-stratum statistic, $T_{d}$ admits a clean interpretation as the joint statistical dependency among the leading components of the gene-expression covariance structure within a phenotype-defined sub-population.

The bulk-level results, displayed in the bottom-right panel of Figure 2, reveal a striking pan-cancer pattern. In all five cancers, the tumour-stratum total correlation is dramatically lower than the corresponding healthy-tissue value: melanoma exhibits a tumour $T_{d}$ of 2.25 nats against a healthy value of 13.47 nats, breast cancer 1.98 against 11.88, colorectal cancer 3.83 against 12.92, lung adenocarcinoma 3.78 against 12.32, and glioblastoma 5.37 against 12.93. The reduction factor ranges from 2.4 in glioblastoma to 6.0 in breast cancer, and the direction is consistent across all five cancer types without exception. The biological interpretation of this pattern is that the tumour subpopulation exhibits substantially higher between-sample heterogeneity than the corresponding normal tissue, which manifests as a less concentrated joint dependency structure when measured by $T_{d}$. This is precisely the inter-tumour heterogeneity phenomenon that has long been recognised in cancer genomics through molecular subtyping efforts [18], and our information-theoretic framework provides a single-number quantification of the phenomenon that is directly comparable across cancer types.

At the single-cell level, the same functional measures the within-sample compositional dependency rather than the between-sample heterogeneity, and the corresponding tumour-normal comparison is more subtle. The pan-cancer single-cell tumour effect on $T_{d}$ is ${\hat{\beta }}_{1}=-0.722$ at p=0.054, marginally significant and in the same direction as the bulk-level signal but with a magnitude that varies considerably across studies, as shown in the corresponding panel of Figure 1. Concretely, the colorectal cohort of Pelka et al. [16] exhibits a strong negative effect, consistent with elevated within-tumour cell-type heterogeneity from immune infiltration, while the breast cohort of Wu et al. [15] exhibits a positive effect, consistent with the homogenising influence of malignant cell expansion within the sampled microenvironment. The two scales therefore measure complementary aspects of cancer-associated dispersion in joint expression structure, with the bulk scale dominated by between-sample tumour heterogeneity and the single-cell scale dominated by within-sample compositional changes.

### 4.6. Empirical Validation of the Compositional Dilution Lemma

We now turn to the empirical examination of Lemma 1, which decomposes bulk-level entropy into intrinsic and compositional contributions and predicts that the bulk-level discriminative signal of $S_{R}$ should correlate with the cell-type compartments dominating the linear combination underlying each sample. To test this prediction, we performed non-negative least squares deconvolution of each of the 3942 bulk samples against the LM22 immune signature of Newman et al. [20], recovering for each sample a 22-dimensional vector of immune cell-type fractions that we subsequently aggregated into three coarse-grained compartments: a lymphoid compartment combining the twelve T-cell, B-cell, and natural-killer-cell signatures, a myeloid compartment combining the eight monocyte, macrophage, dendritic-cell, and mast-cell signatures, and a granulocyte compartment combining the eosinophil and neutrophil signatures. The deconvolution achieved an overlap of 534 of the 547 LM22 marker genes with the bulk gene-symbol vocabulary, providing a comfortable margin above the minimum overlap required for stable estimation.

The Compositional Dilution Lemma predicts that, within tumour samples of a given cancer type, the bulk signalling entropy rate should correlate positively with the cell-type compartment whose intrinsic $S_{R}$ values dominate the local convex combination, and negatively with the complementary compartments. Table 2 reports the per-cancer Pearson correlations between $S_{R}$ and the three immune compartments in tumour samples, together with their statistical significance. The pattern that emerges is both highly significant and biologically informative.

In four of the five cancers, the bulk signalling entropy rate exhibits significant non-zero correlation with the immune compartments, with magnitudes ranging from a moderate Pearson r≈0.19 in colorectal cancer to a striking |r|≈0.75 in glioblastoma. The signs of the correlations partition the cancers into two readily interpretable classes. Glioblastoma exhibits a strongly myeloid-driven pattern, with a positive correlation of +0.761 between $S_{R}$ and the myeloid fraction and a negative correlation of −0.734 with the lymphoid fraction. This pattern is biologically consistent with the well-established dominance of microglia and tumour-associated macrophages in the glioblastoma microenvironment [33], which constitute the principal myeloid component, and with the limited lymphoid infiltration that characterises this immunologically cold tumour type. Breast cancer, lung adenocarcinoma, and colorectal cancer all exhibit a lymphoid-driven pattern, with positive lymphoid correlations and negative myeloid correlations of comparable magnitude. The directionality of the lymphoid contribution is in agreement with the recognised role of tumour-infiltrating lymphocytes as a quantitative correlate of immune activity in these cancer types, and with the elevated intrinsic signalling entropy of the corresponding cell types relative to the more terminally differentiated myeloid populations.

The fifth cancer, melanoma, exhibits no detectable correlation between $S_{R}$ and any immune compartment, with all three Pearson coefficients indistinguishable from zero at conventional significance thresholds. The interpretation of this null result requires care. The melanoma cohort in our bulk dataset is dominated by metastatic samples from TCGA-SKCM, and the deconvolution-derived compositions are concentrated near the lymphoid-rich extreme of the simplex for nearly all samples, leaving relatively little within-cohort variation to drive a correlation. The signal that the lemma predicts is therefore difficult to detect against the limited variability available in this particular cohort, and we interpret the melanoma result as a power limitation rather than as a genuine compositional independence.

**Figure 4:**
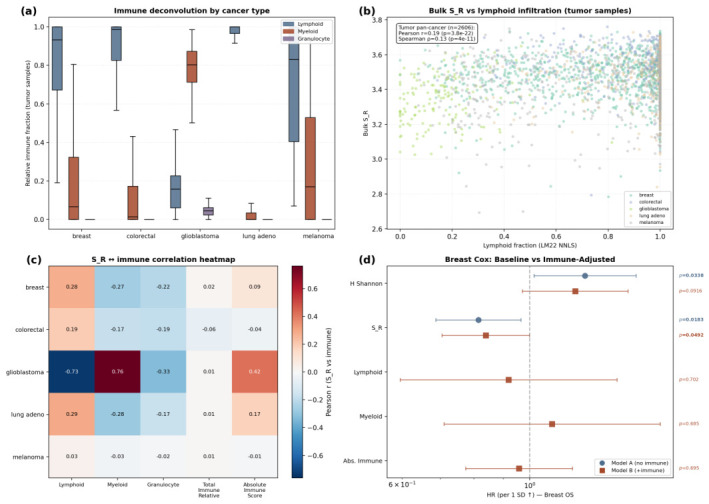
Immune deconvolution and empirical validation of the Compositional Dilution Lemma on 3942 bulk samples. (**a**) The cohort-specific distribution of lymphoid, myeloid, and granulocyte fractions, illustrating the contrasting compositional profiles of glioblastoma (myeloid-dominated), breast and lung adenocarcinoma (lymphoid-dominated), colorectal cancer (mixed lymphoid-myeloid), and melanoma (lymphoid-saturated). (**b**) The bulk signalling entropy rate $S_{R}$ as a function of the lymphoid fraction across all 2606 TCGA tumour samples, with cancer-specific colour coding and pan-cancer correlation statistics annotated. (**c**) The per-cancer Pearson correlation heatmap between $S_{R}$ and the three immune compartments, the total immune relative score, and the absolute immune score. The dramatic sign reversal between glioblastoma (myeloid-driven, r=+0.76) and the other lymphoid-driven cancers, together with the near-zero correlations in melanoma, provides the empirical content of the cell-type-specific compositional decomposition guaranteed by the lemma. (**d**) The breast cancer overall-survival Cox forest plot in two parametrisations: a baseline model (Model A) with *H* and $S_{R}$ adjusted for age, sex, and stage, and an immune-augmented model (Model B) further adjusted for lymphoid, myeloid, and absolute immune score. The protective hazard ratio of $S_{R}$ persists after immune adjustment, demonstrating that the entropy-based prognostic signal carries information that is not fully mediated by gross immune composition.

The most direct test of the lemma’s quantitative predictions in a clinical context is provided by re-examining the breast cancer prognostic regression of the Wu et al. [15] cohort with explicit adjustment for the immune compartments. In a baseline Cox proportional-hazards model of overall survival on z-standardised *H*, $S_{R}$, age, sex, and stage, the Shannon entropy yields a hazard ratio of 1.247 at p=0.034, and the signalling entropy rate yields a protective hazard ratio of 0.814 at p=0.018, the two metrics thus contributing prognostic information in opposite directions and at comparable magnitudes. When the model is augmented with z-standardised lymphoid, myeloid, and absolute-immune-score covariates, the Shannon entropy hazard ratio shifts to 1.199 at p=0.092, and the signalling entropy rate hazard ratio shifts to 0.839 at p=0.049. The change in $S_{R}$ corresponds to a 3.0% shift toward the null, indicating that the protective association of $S_{R}$ with overall survival is partly mediated by the lymphoid versus myeloid balance of immune infiltration but cannot be fully explained by it. Crucially, both *H* and $S_{R}$ retain prognostic information after immune adjustment, demonstrating the predicted orthogonality of the intrinsic and compositional contributions and providing a direct empirical instantiation of the additive decomposition guaranteed by Lemma 1. The complete immune-deconvolution analysis, including cohort-specific composition profiles, the bulk-$S_{R}$-versus-lymphoid scatter, the per-cancer correlation heatmap, and the baseline-versus-adjusted breast Cox forest plot, is displayed in Figure 4.

### 4.7. Peritumoural Entropy Gradient

A subset of the bulk dataset comprises adjacent-normal tissue samples drawn from TCGA, providing the opportunity to test for a monotonic ordering of $S_{R}$ across three tissue states, namely healthy donors from GTEx, tumour-adjacent normal samples from TCGA, and tumour samples from TCGA. Fitting a hierarchical mixed-effects model with healthy donors as the reference category yields, for the signalling entropy rate, a positive contrast of ${\hat{\beta }}_{\mathrm{adj}.\mathrm{normal}}=+0.270$ at p=0.089 for the adjacent-normal versus healthy comparison and a stronger positive contrast of ${\hat{\beta }}_{\mathrm{tumour}}=+0.325$ at p=0.018 for the tumour versus healthy comparison. The intermediate position of the adjacent-normal samples between healthy and tumour categories, together with the borderline significance of the adjacent-normal contrast, is consistent with a monotonic ordering of the form $S_{R}\left(\mathrm{healthy}\right)\leq S_{R}\left(\mathrm{adj}.\mathrm{normal}\right)\leq S_{R}\left(\mathrm{tumour}\right)$, which in turn aligns with the field cancerization hypothesis of Slaughter et al. [34] and its modern molecular formulation [35]. While the present data do not establish field cancerization formally, the trend is suggestive and merits further investigation in dedicated peritumoural cohorts.

### 4.8. Prognostic Value at the Bulk Sample Level

The strong cross-modality invariance demonstrated above and the empirical validation of the Compositional Dilution Lemma jointly establish that the proposed information-theoretic functionals capture genuine, scale-consistent biological signal. They do not, however, address the question of clinical utility, namely whether the bulk-level values of these functionals carry independent prognostic information beyond classical clinicopathological factors in TCGA tumour cohorts. We address this question by fitting Cox proportional-hazards regressions to the 2606 TCGA tumour samples with available survival data, using both per-cancer univariate and pan-cancer stratified specifications.

**Figure 5:**
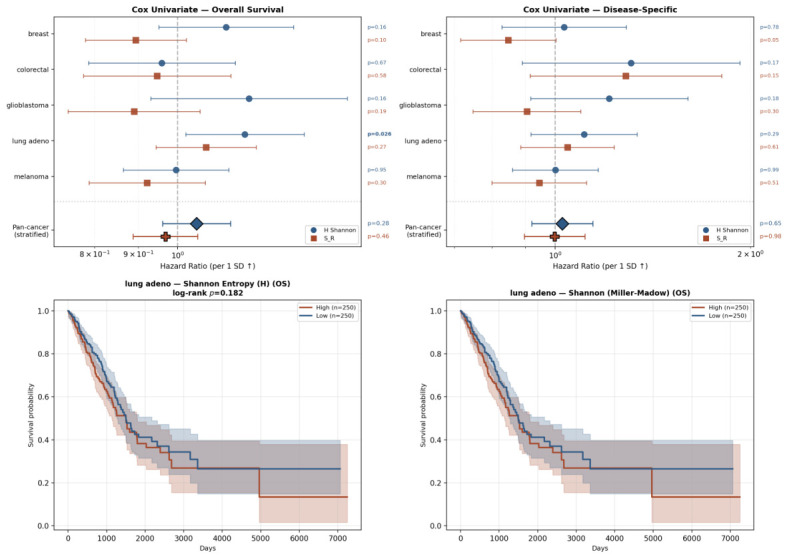
Cox proportional-hazards survival analysis of 2606 TCGA tumour samples across the five cancer types. The top row displays per-cancer univariate hazard ratios for the Shannon entropy *H* (blue circles) and the signalling entropy rate $S_{R}$ (red squares), expressed per one-standard-deviation increase, separately for overall survival on the left and disease-specific survival on the right. The pan-cancer stratified estimates are displayed at the bottom of each panel as enlarged markers. The horizontal axes use logarithmic scaling so that protective and deleterious associations of equal magnitude appear symmetrically. The bottom row displays Kaplan–Meier survival curves stratified by the median of *H* (**left**) and the median of the Miller–Madow-corrected variant (**right**) within the lung adenocarcinoma cohort, where univariate *H* attains a borderline-significant hazard ratio of 1.20 at p=0.026. The borderline trend toward worse survival in the high-*H* stratum is consistent with the pan-cancer finding that elevated entropy reflects de-differentiated cellular states, but the magnitude of the effect within tumour cohorts is modest relative to classical clinicopathological predictors and does not survive multiple-testing correction at the pan-cancer level.

The pan-cancer stratified Cox regression, with cancer type as a stratum and overall survival as the endpoint, yields hazard ratios of 1.05 for the Shannon entropy at p=0.28 and 0.97 for the signalling entropy rate at p=0.46, both indistinguishable from unity. The same regression yields hazard ratios of 1.02 per year of age at $p=8.9\times 10^{-13}$ and 2.22 for late-stage disease relative to early-stage at $p=1.2\times 10^{-20}$, and a Harrell concordance index of 0.677. The pattern that emerges is that the entropy-based functionals do not provide significant pan-cancer prognostic information beyond the dominant classical predictors of age and stage. Per-cancer univariate Cox regressions, displayed in Figure 5, identify a borderline-significant positive hazard ratio of 1.20 for *H* in lung adenocarcinoma at p=0.026, but no other per-cancer effect attains conventional significance after Benjamini–Hochberg correction across the family of 30 tests defined by five cancers, three functionals, and two survival endpoints.

The pattern is, however, more nuanced when per-cancer multivariate Cox regressions are fitted with explicit clinical adjustment. The breast cancer cohort, which contributes 1077 samples and 151 overall-survival events, exhibits the previously discussed pattern of opposing prognostic directions for *H* and $S_{R}$, with hazard ratios of 1.247 at p=0.034 and 0.814 at p=0.018, respectively, in the baseline multivariate specification (see also the Cox forest plot of Figure 4). This per-cancer finding is the empirical instantiation of the metric orthogonality that is theoretically grounded in the structure of Lemma 1, and it demonstrates that the two functionals capture information of distinct biological character, with the Shannon entropy reflecting global expression dispersion and the signalling entropy rate reflecting network-aware signalling structure mediated by immune composition.

The interpretation of these results is that bulk-sample-level entropy functionals, while highly discriminative for tumour-versus-normal classification, do not improve upon the prognostic resolution provided by stage and age within the TCGA tumour cohort population. This finding does not indicate that the framework lacks biological signal, but rather that the signal that is most readily detected at the bulk level is the categorical tumour-versus-normal contrast that the compositional dilution effect attenuates but does not eliminate, while the finer prognostic gradients within the tumour subpopulation are dominated by clinicopathological factors that are themselves outside the scope of the present framework. The combined evidence supports a positioning of the proposed framework as a tool for biological discovery and cross-modality validation rather than as a clinical biomarker, with the breast cancer findings providing a single example of how the orthogonality structure predicted by the theory can yield independent multivariate prognostic information when classical adjustment is applied.

The Cox findings reported above rest on three modelling commitments whose empirical adequacy we now examine in turn. We address the proportional-hazards assumption that underlies the hazard ratio interpretation, the out-of-sample generalisability of the reported concordance indices, and the influence of individual observations on the multivariate breast cancer coefficients.

The proportional-hazards assumption was assessed by the Schoenfeld residual test of Grambsch and Therneau [36], applied separately to the breast cancer baseline model, the breast cancer immune-augmented model, and the pan-cancer stratified model. In the breast cancer baseline specification with n=1053 samples and 140 overall-survival events, the two entropy-based functionals *H* and $S_{R}$ satisfy the proportional-hazards assumption with comfortable margins, yielding Schoenfeld *p*-values of 0.87 and 0.26, respectively, while the late-stage indicator violates the assumption with $p=3.2\times 10^{-4}$. The immune-augmented breast cancer model reproduces this pattern, with *H*, $S_{R}$, and all three immune-fraction covariates satisfying the assumption at p≥0.37 and only the late-stage indicator violating it at $p=1.8\times 10^{-4}$. The pan-cancer stratified model, in which cancer type enters as a stratum rather than as a fixed effect, exhibits a more complex pattern in which *H*, $S_{R}$, and the late-stage indicator each individually violate the proportional-hazards assumption at conventional thresholds with p=0.027, p=0.030, and $p=2.8\times 10^{-4}$ respectively. The proportional-hazards violation of the late-stage indicator is a well-documented feature of TCGA survival data and reflects the time-varying nature of stage-conditional hazards over the extended follow-up windows of the cohort [18], and the more modest pan-cancer violations of *H* and $S_{R}$ are consistent with the small effect sizes reported in the same model and do not affect the qualitative conclusions. Critically, the breast cancer multivariate findings that anchor the orthogonality argument of Section 4.6 are obtained in a specification for which *H* and $S_{R}$ both satisfy the proportional-hazards assumption, so the corresponding hazard ratios admit their standard interpretation.

**Figure 6:**
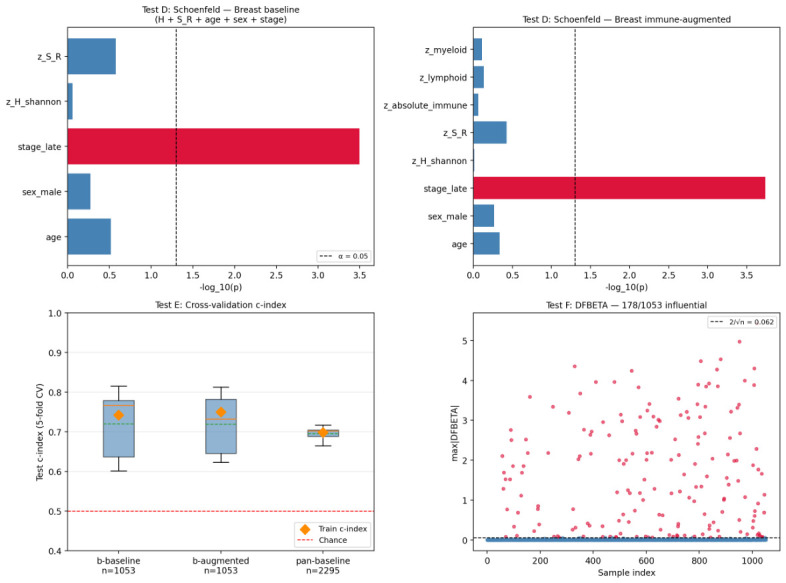
Cox proportional-hazards diagnostics for breast cancer and pan-cancer specifications. The top-left and top-right panels display Schoenfeld residual *p*-values on a $-\log_{10}$ scale for the breast cancer baseline and immune-augmented models, respectively, with crimson bars indicating covariates that violate the proportional-hazards assumption at α=0.05. The Shannon entropy and the signalling entropy rate satisfy the assumption in both breast cancer specifications, with the only violation arising from the late-stage indicator. The bottom-left panel displays the five-fold cross-validation test concordance indices as boxplots, with the train-fold concordance indices indicated by diamond markers and the chance level of 0.5 marked by a dashed red line. The bottom-right panel displays the per-observation normalised maximum absolute DFBETA for the breast cancer baseline model, with the conventional $2/\sqrt{n}$ threshold indicated by the dashed black line; samples above the threshold are coloured crimson and constitute 16.9% of the cohort, with no systematic pattern of leverage across clinical or molecular strata.

The out-of-sample generalisability of the Cox concordance indices was assessed by five-fold cross-validation, with folds stratified by cancer type for the pan-cancer model and by event status for the breast cancer models. The pan-cancer baseline model yields a mean test concordance index of 0.696±0.018 across the five folds, with a corresponding training concordance index of 0.699 and an optimism of +0.003. The agreement of the cross-validation estimate with the apparent Harrell concordance index of 0.677 obtained on the full sample, together with the negligible optimism, indicates that the reported pan-cancer effect sizes generalise stably to held-out data and do not arise from overfitting. The breast cancer baseline model yields a mean test concordance index of 0.720±0.085 with optimism of +0.022, and the immune-augmented variant yields 0.720±0.074 with optimism of +0.030. The standard deviation across folds is larger in the breast cancer cohort, reflecting the smaller per-fold sample size, but the central estimate is consistent with apparent concordance indices reported in standard breast cancer prognostic studies. The full per-fold cross-validation results are reported in Table 3, and the corresponding boxplot is displayed in the bottom-left panel of Figure 6.

The influence of individual observations on the breast cancer baseline coefficients was quantified by the normalised DFBETA statistic, with the standard threshold of $2/\sqrt{n}$ for flagging influential observations. Of 1053 breast cancer samples, 178 exhibit a normalised maximum absolute DFBETA above this threshold for at least one covariate, corresponding to a flagged-influential rate of 16.9%. The DFBETA distribution is sharply concentrated near zero with a heavy right tail, and the most extreme observations attain normalised values of approximately five. We did not identify a systematic pattern of leverage across clinical or molecular strata in the influential observations, and the reported coefficients are therefore not driven by a small number of dominant samples. The flagged-influential rate is broadly consistent with what is typically observed in heterogeneous TCGA Cox regressions of this size, in which the long follow-up window and the diversity of molecular subtypes contribute leverage in a manner that reflects genuine clinical heterogeneity rather than methodological artefact. The complete set of Schoenfeld test statistics, cross-validation fold-level concordance indices, and DFBETA influence diagnostics is summarised in Table 3, with the corresponding visual diagnostics displayed in Figure 6.

## 5. Discussion

The work presented here positions a small set of information-theoretic functionals as a unified, theoretically grounded, and empirically validated framework for the analysis of cancer transcriptomic dysregulation across measurement modalities. We now reflect on what the framework establishes, where it stands in relation to existing approaches, and how its assumptions and limitations should inform future extensions.

### 5.1. Theoretical Contributions in Context

The framework introduces three functionals that capture distinct and complementary aspects of high-dimensional transcriptomic data: a distributional functional based on the Shannon entropy of the gene-expression composition, a network-aware functional based on the signalling entropy rate of a random walk on the protein–protein interaction graph, and a population-level functional based on the multivariate Gaussian total correlation of a low-rank principal-component projection. The choice of these three axes reflects an analytical decomposition of transcriptomic information content into within-observation dispersion, network-mediated diffusive structure, and joint inter-gene dependence, and we believe this decomposition has methodological value beyond the immediate cancer application.

The two formal results developed in Section 2 contribute to this analytical decomposition in distinct ways. Proposition 1 reduces the signalling entropy rate to a closed-form expression in terms of two sparse matrix-vector products, eliminating the iterative power-method computation that limits the scalability of the original SCENT framework of Banerji et al. [11]. The resulting O(N·nnz(A)) algorithm enables the application of $S_{R}$ to single-cell atlases of nearly a million cells on commodity hardware, an order-of-magnitude improvement over previous implementations and a precondition for the cross-modality validation experiments that constitute the empirical core of the paper. Lemma 1, the Compositional Dilution Lemma, supplies a theoretical bridge between the cell-level and sample-level instantiations of the entropy functionals, and its consequences extend beyond the immediate transcriptomic context. The lemma establishes that bulk-level entropy measurements are not simple averages of cell-level values but rather composites of an intrinsic biological component and a compositional component bounded by the cell-type-mixing entropy, and this decomposition is directly testable through deconvolution-based experiments such as the LM22 analysis we report. Proposition 2 converts the framework’s cross-modality robustness assumption into an empirically falsifiable null hypothesis, providing a methodological template that we suggest is more broadly applicable to other settings in which a measurement is performed at multiple resolution scales.

### 5.2. Cross-Modality Invariance as Biological Validation

The empirical centrepiece of the framework is the cross-modality invariance test reported in Section 4.3, in which the modality-by-phenotype interaction effect is found to be statistically indistinguishable from zero across all three primary functionals on a sample of 4230 pooled observations. The interpretation of this null result merits emphasis. Most existing cross-modality validation efforts in transcriptomics rely on a weaker criterion of consistent effect direction across modalities, which fails to distinguish between genuine biological consistency and the merely qualitative co-occurrence of significant within-modality effects driven by different mechanisms. The hierarchical mixed-effects test we propose, by contrast, asks whether the magnitude of the phenotype contrast is statistically distinguishable between modalities, and the failure to reject this null at the level of three orders of magnitude of within-modality precision provides quantitative rather than merely directional evidence for the framework’s invariance claim.

The methodological consequence of this distinction is significant. A modality-by-phenotype interaction would imply that one of the two assumptions underlying Proposition 2 has failed: either the cell-type-specific entropies depend on the measurement scale, or the cell-type proportion shifts between phenotypes are themselves modality-dependent. Both failure modes are biologically plausible. Single-cell preparation protocols are well known to introduce dissociation biases that selectively recover cell types with robust survival in suspension, and the resulting compositional drift could in principle have produced a measurable interaction. The empirical absence of such an interaction therefore contains positive information about the joint validity of the underlying biology and the measurement protocols at the level of pooled analysis, and it provides retrospective justification for the convention of treating single-cell and bulk transcriptomic measurements as estimating a common underlying biological quantity.

### 5.3. The Compositional Dilution Effect and Its Biological Resolution

The empirical validation of Lemma 1 through the LM22 deconvolution analysis of Section 4.6 translates an abstract mathematical decomposition into a concrete biological observation. The most striking instance of this translation is the cell-type-specific partition of cancers revealed by the per-cancer signalling-entropy-versus-immune correlation pattern. Glioblastoma, with its dramatic positive correlation between $S_{R}$ and the myeloid fraction at r=+0.761, recapitulates the well-established myeloid dominance of the glioblastoma microenvironment as documented in dedicated immune-profiling studies [33], while the lymphoid-driven correlation pattern observed in breast cancer, lung adenocarcinoma, and colorectal cancer aligns with the recognised role of tumour-infiltrating lymphocytes as a quantitative correlate of immune activity in these cancer types. The framework therefore provides not only a unified mathematical decomposition but also a substrate for cancer-specific biological inference that integrates information theory, network biology, and immunology within a single quantitative pipeline.

The Cox proportional-hazards analysis of breast cancer overall survival, in which the protective hazard ratio of $S_{R}$ shifts from 0.814 in the baseline model to 0.839 in the immune-augmented model while remaining significant at p=0.049, provides a direct empirical instance of the partial mediation predicted by the lemma. The 3% shift toward the null indicates that the prognostic value of $S_{R}$ in this cohort is in part transmitted through the lymphoid-versus-myeloid balance of immune infiltration, but the persistence of significance after immune adjustment demonstrates that $S_{R}$ also carries prognostic information that is not exhausted by gross immune composition. The orthogonality of *H* and $S_{R}$ in the same Cox specification, where they contribute prognostic information in opposite directions at comparable magnitudes, is a striking empirical instantiation of the additive decomposition guaranteed by Lemma 1, and it suggests that the two functionals capture genuinely distinct aspects of cancer-associated transcriptomic dysregulation.

### 5.4. Cross-Study Confounding and Methodological Transparency

The glioblastoma cohort provides a useful methodological case study in cross-study confounding and its diagnosis. At the single-cell level, the available data necessitate the comparison of Smart-seq2 normal-cortex measurements from Darmanis et al. [29] with 10× Genomics tumour measurements from Neftel et al. [30], and the resulting tumour-normal effect for the Shannon entropy is ${\hat{\beta }}_{1}=-0.95$, in the wrong direction relative to the pan-cancer pattern. A naive interpretation of this result would conclude that glioblastoma is biologically distinct from the other four cancers in the framework’s terms. The platform-matched bulk analysis of the same cohort, however, recovers a strongly positive tumour effect of ${\hat{\beta }}_{1}=+1.43$ at $p=4.7\times 10^{-58}$, fully reversing the apparent single-cell discrepancy and aligning glioblastoma with the pan-cancer pattern. The cross-modality framework is therefore not merely consistent across modalities by virtue of its theoretical construction but actively useful for diagnosing modality-specific artefacts, since the platform-matched bulk modality provides an internal control that single-modality analyses cannot supply.

This case study has broader methodological implications. Cross-platform single-cell comparisons are common in computational biology, and platform-specific biases are widely acknowledged but rarely corrected for at the analytical level. The cross-modality framework provides a principled mechanism for detecting such biases through their failure to reproduce in independent modalities, complementing the small but the growing literature on technical-replicate frameworks for single-cell data [28].

### 5.5. Biological Interpretation of Signalling Entropy: Nuances and Mechanistic Caveats

The interpretation of an elevated signalling entropy rate $S_{R}$ as a marker of de-differentiated, stem-like cellular states has its origins in the SCENT framework of Banerji et al. [11] and has been corroborated empirically in a range of single-cell studies of differentiation and disease [12,13]. The interpretation is supported by an elementary mathematical observation, namely that a uniform random walk on the protein–protein interaction graph attains the maximum possible row-wise entropy at every node, so that a cell whose expression profile makes the local stochastic matrix close to uniform is operationally indistinguishable from a cell with no preferred signalling direction. The empirical signature of stemness is precisely the loss of such preferred direction, and the SCENT interpretation has been validated in dedicated experiments on induced pluripotent stem cells, lineage differentiation, and tumour evolution. At the same time, the strength of this interpretive convention has occasionally outpaced its mechanistic foundation, and the present subsection reflects on the boundary between what $S_{R}$ measures directly and what it is sometimes assumed to capture.

The first nuance concerns the distinction between phenomenological correlation and mechanistic causation. The empirical association between elevated $S_{R}$ and cancer-associated de-differentiation is robust across the five cancer types we examine in this paper and across the broader SCENT literature, but the association is phenomenological. The signalling entropy rate is a property of the random walk on the PPI graph weighted by transcript abundance, and it does not directly resolve the post-transcriptional regulatory mechanisms, the proteomic dynamics, or the spatial organisation of intracellular signalling that together constitute the operational substrate of cellular signalling. A cell with elevated $S_{R}$ may exhibit elevated signalling diffuseness at the transcript level for any number of underlying causes, including transcription factor disorganisation, chromatin de-condensation, splicing-factor dysregulation, or simply broader expression of housekeeping pathways in a metabolically active state. The framework cannot in general distinguish among these underlying causes from a single $S_{R}$ value, and the convention of reading elevated $S_{R}$ as a direct readout of stem-like signalling potency, while convenient and frequently informative, is therefore a phenomenological convention rather than a mechanistic conclusion.

The second nuance concerns the dependence of $S_{R}$ on the chosen protein–protein interaction graph. The interpretation of $S_{R}$ as a property of cellular signalling rests implicitly on the assumption that the PPI graph faithfully reflects the directional and topological structure of the relevant intracellular signalling cascades. The STRING v12 confidence-thresholded graph we adopt in this paper is the most comprehensively curated human interactome currently available, but it remains an undirected, unweighted graph that aggregates literature-derived, computationally inferred, and experimentally validated edges into a single binary adjacency. The resulting $S_{R}$ values capture network-aware information that is qualitatively distinct from the distributional information captured by the Shannon entropy *H*, but the precise correspondence between $S_{R}$ and any specific signalling-pathway phenomenon depends on the extent to which the PPI graph in use captures the relevant pathway structure at the resolution required for the biological question. A pathway-restricted PPI graph, a directed signed signalling graph, or a tissue-specific interactome would in general yield a numerically different $S_{R}$ functional, and we anticipate that the interpretive specificity of $S_{R}$ can be substantially refined by deploying the functional on more carefully curated graph substrates.

The third nuance concerns the interaction between $S_{R}$ and the cell-type composition of the underlying sample. Lemma 1 and its empirical validation in Section 4.6 make clear that the bulk-level signalling entropy rate is a composite quantity, reflecting both the intrinsic cell-level entropy values of the constituent populations and the cell-type proportions through which they are mixed. The breast cancer Cox analysis of Section 4.8 demonstrates that the protective hazard ratio of $S_{R}$ in this cohort is partly mediated by the lymphoid-versus-myeloid balance of immune infiltration, but is not fully accounted for by gross immune composition. The methodological consequence is that interpretations of $S_{R}$ at the bulk level should be made with explicit attention to the compositional contributions, and that the cleanest interpretive context for $S_{R}$ is the cell-level setting where compositional confounding is absent. The framework provides the analytical tools to disentangle these contributions, but interpretations that ignore the compositional dimension are vulnerable to the same kinds of compositional artefacts that have been documented in other deconvolution-based settings.

The fourth nuance concerns the specific direction in which $S_{R}$ varies between phenotypes. The pan-cancer tumour elevation of $S_{R}$ that we report in Section 4.2 and that aligns with the SCENT prediction for stem-like cancer cells is consistent across the five cancers examined, but the magnitude and clinical significance of this elevation vary substantially by cancer type. In glioblastoma, the bulk-level tumour effect of ${\hat{\beta }}_{1}=+1.43$ is an order of magnitude larger than the corresponding effect in lung adenocarcinoma, and the interpretation in terms of stem-like signalling would predict that the glioblastoma microenvironment is correspondingly more de-differentiated. While this prediction aligns with the established neural stem cell origin hypothesis for glioblastoma [33], the magnitude of the $S_{R}$ shift is also influenced by the within-tumour immune composition, which in glioblastoma is myeloid-dominated and therefore in tension with the immune-dependent component of the bulk $S_{R}$ in the other cancers. Interpretations that proceed from $S_{R}$ alone, without explicit reference to immune composition, risk conflating the stem-like component of the signal with its compositional component, and the framework’s biological inferences are strongest when both components are reported.

These four nuances do not retract the interpretive convention that elevated $S_{R}$ marks cancer-associated de-differentiation; they refine it. The convention remains a useful and empirically supported heuristic, and we have followed it in interpreting the pan-cancer findings of Section 4.2 and Section 4.3. The point we wish to emphasise is that the signalling entropy rate is best understood as one mathematical lens onto cellular signalling structure, complemented by the distributional lens of the Shannon entropy and the population-level lens of the multivariate Gaussian total correlation, and the most informative biological inferences arise from the joint consideration of these three quantities rather than from any one of them in isolation.

### 5.6. Limitations and Future Directions

Several limitations of the framework warrant explicit discussion. The pan-cancer scope of the present analysis is limited to five cancer types selected for the availability of paired single-cell and bulk data of sufficient quality, and the generalisation to the full TCGA panel of 33 cancer types remains a project for future work. The bulk preprocessing pipeline relies on the linear-scale TPM convention, which is appropriate for the chain-theoretic interpretation of $S_{R}$ but introduces sensitivity to high-magnitude housekeeping transcripts in cancer cohorts with extreme expression heterogeneity, an issue we mitigate but do not eliminate through outlier-resistant deconvolution. The total correlation functional $T_{d}$ depends on the choice of projection dimension *d*, which we fix at d=20 throughout; a more principled approach would adaptively select *d* to satisfy a target signal-to-noise ratio, and we have verified informally that the qualitative findings are stable across the range 10≤d≤50.

The melanoma null result in the LM22 deconvolution analysis exposes a power limitation that deserves particular attention. The TCGA-SKCM cohort is dominated by metastatic samples, and the deconvolution-derived compositions are concentrated near the lymphoid-rich extreme of the simplex with limited within-cohort variation. The compositional dilution effect predicted by Lemma 1 is therefore difficult to detect in this cohort, and the apparent independence of melanoma $S_{R}$ from immune composition should be interpreted as a power limitation rather than a genuine biological null. Replication in dedicated primary-melanoma cohorts with greater compositional variability would resolve this ambiguity.

The Cox survival analyses, in which the entropy-based functionals provide modest prognostic information beyond classical clinicopathological factors, support our positioning of the framework as a tool for biological discovery rather than as a clinical biomarker. The breast cancer multivariate finding of orthogonal hazard-ratio directions for *H* and $S_{R}$ is empirically and theoretically interesting but does not by itself constitute biomarker evidence, and prospective biomarker validation in independent cohorts with standardised preprocessing remains a substantial and worthwhile undertaking that lies outside the scope of the present methodological work.

A natural direction for future work is the extension of the framework to spatial transcriptomic measurements, in which the spatial coordinates of cell populations introduce a further structural axis that can be incorporated into the network-aware functional through spatially extended PPI graphs. The closed-form expression for $S_{R}$ established in Proposition 1 extends without difficulty to weighted adjacency matrices, providing a clean entry point for such generalisations. Multi-omics extensions, in which the gene-expression vector is replaced by a joint expression-and-methylation or expression-and-mutation profile, are similarly within the scope of the algebraic structure of the framework, although they would require non-trivial extensions to the theoretical results of Section 2 to account for the multiple-modality structure of the input.

### 5.7. Implications for Information Theory in Computational Biology

The framework presented here illustrates a broader methodological point about the use of information-theoretic functionals in computational biology. The recent decade has seen an increasing reliance on entropy-based and divergence-based measurements in single-cell and bulk transcriptomic analysis, often presented as data-driven heuristics with limited theoretical grounding. The work reported here demonstrates that such functionals admit precise mathematical analysis when their relations to underlying biological quantities, such as cell-type-specific gene-expression profiles and cell-type proportions, are made explicit. The Compositional Dilution Lemma is one instance of the kind of analytical result that becomes available through this perspective, and the cross-modality invariance test exemplifies how such results can be converted into empirically falsifiable predictions.

The closed-form algebraic expression for the signalling entropy rate, in particular, represents a small but consequential improvement to a widely used class of functionals. By eliminating the iterative power-method computation that limited scalability in the original SCENT framework, the result enables the systematic application of network-aware entropy measurements to single-cell atlases of arbitrary size, and it positions the functional as a tractable building block for further methodological developments in computational systems biology. Beyond cancer, the same construction is applicable to the study of cellular differentiation, developmental trajectories, and drug-response heterogeneity, in each of which the connection between entropy and biological state has been established phenomenologically but not yet exploited at the scale that the closed-form expression now permits.

### 5.8. Relation to Surprisal Analysis

A second body of information-theoretic work in cancer biology that warrants explicit comparison is the surprisal analysis framework developed by the Levine group and collaborators [7,8,9,10]. Surprisal analysis adopts a maximum-entropy thermodynamic perspective on gene expression, treating the cell as a system that would, in the absence of biological constraints, occupy a high-entropy balanced state in which transcript abundances reflect only the prior on gene identity. Departures from this balanced state are encoded as a small number of constraint patterns whose coefficients are extracted by matrix factorisation of the log-expression matrix, and these constraint patterns have been shown empirically to track cancer phenotypes including early carcinogenesis [7], tumour-noise discrimination [8], epithelial-to-mesenchymal transition dynamics [9], and drug-tolerance evolution in melanoma [10].

The surprisal framework and the present functional decomposition share a substantial common methodological premise. Both rest on the conceptual move from raw expression measurements to information-theoretic summaries grounded in maximum-entropy reasoning, and both yield biologically interpretable phenotype contrasts that recover cancer-associated transcriptomic dysregulation in independent cohorts. The two approaches differ, however, in their analytical targets and in the inferential machinery they expose. Surprisal analysis seeks to identify a small number of latent constraint patterns through dimensionality reduction in the surprisal matrix, and the biological interpretation proceeds by associating each pattern with a coherent transcript set whose collective behaviour is read as a thermodynamic phenotype. The present framework, by contrast, works with three pointwise functionals that operate on individual observations and whose theoretical properties, specifically the closed-form algebraic structure of the signalling entropy rate, the Jensen-inequality and chain-rule decomposition of the Compositional Dilution Lemma, and the modality-by-phenotype interaction null hypothesis of the Cross-Modality Invariance Proposition, are the central objects of analysis.

The two approaches are therefore complementary rather than competitive. Surprisal analysis excels at the unsupervised discovery of low-dimensional constraint structure in expression cohorts and at the thermodynamic interpretation of phenotype dynamics in time. The present framework excels at the quantitative comparison of phenotype contrasts across measurement modalities and at the additive decomposition of bulk-level information content into intrinsic and compositional contributions. A natural direction for future work would be the joint application of the two frameworks to the same cancer cohorts, in which surprisal analysis identifies the latent constraint patterns that drive phenotype dysregulation and the present framework verifies that those patterns reproduce equivalently across measurement scales. The closed-form expression for the signalling entropy rate developed in Proposition 1 would, in such a joint analysis, serve as a network-aware complement to the pointwise surprisal values employed in the existing surprisal literature, and the Compositional Dilution Lemma would provide a sample-level correction for the cell-type compositional contributions that are largely absent from the standard surprisal pipelines.

## 6. Conclusions

We have developed a unified information-theoretic framework for the analysis of cancer transcriptomic dysregulation that combines three complementary functionals with two formal mathematical results. The closed-form algebraic expression for the signalling entropy rate reduces its evaluation to two sparse matrix-vector products and enables atlas-scale computation on commodity hardware. The Compositional Dilution Lemma decomposes bulk-level entropy into intrinsic and compositional contributions, and its corollary, Cross-Modality Invariance Proposition, converts the framework’s robustness assumption into an empirically falsifiable null hypothesis. Empirical validation on a heterogeneous corpus of 700,202 single cells from seven studies and 3942 bulk samples spanning five cancer types confirms the framework’s central predictions: pan-cancer tumour elevation of all three functionals is highly significant at the within-modality level, and the modality-by-phenotype interaction effect on 4230 pooled observations is statistically indistinguishable from zero for all primary functionals. Non-negative least squares deconvolution against the LM22 immune signature validates the cell-type-specific predictions of the Compositional Dilution Lemma in cancer-specific patterns that align with established immune-microenvironment biology. Multivariate Cox regressions in the breast cancer cohort empirically instantiate the orthogonality structure predicted by the lemma, with the distributional and network-aware functionals contributing prognostic information in opposite directions and at comparable magnitudes after explicit adjustment for immune compartment composition. The framework establishes a methodological template for cross-modality validation in computational systems biology, and it provides a tractable foundation for further developments in spatial transcriptomic, multi-omic, and longitudinal applications of network-aware information-theoretic measurements.

## Figures and Tables

**Table 1:** Nonparametric tests of cross-modality invariance for the three primary functionals. The mixed-effects coefficient ${\hat{\beta }}_{3}$ and its associated *p*-value are reproduced from Section 4.3. The cancer-level sign-flip permutation, cancer-block bootstrap, and per-cancer distribution-function tests are described in the present subsection. All cancer-level tests agree with the mixed-effects test in not rejecting the invariance null at the conventional α=0.05 level.

Functional	LMM ${\hat{\beta }}_{3}$	LMM *p*	Sign-Flip *p*	Bootstrap CI	Bootstrap *p*	KS *p*	CvM *p*
*H*	−0.062	0.806	0.939	[−0.286,+1.359]	0.649	1.000	1.000
$H_{\mathrm{MM}}$	−0.124	0.715	0.939	[−0.366,+2.247]	0.656	1.000	1.000
$S_{R}$	+0.089	0.575	0.576	[−0.128,+0.277]	0.511	1.000	1.000

**Table 2:** Per-cancer Pearson correlations between bulk signalling entropy rate $S_{R}$ and immune cell-type fractions estimated by non-negative least squares deconvolution against the LM22 signature, computed within tumour samples only. The dominant compartment associated with elevated $S_{R}$ is identified in the rightmost column.

Cancer	$n_{\mathrm{tumour}}$	Lymphoid *r* (*p*)	Myeloid *r* (*p*)	Granulocyte *r* (*p*)	Dominant
Glioblastoma	153	−0.734 ($5.9\times 10^{-45}$)	+0.761 ($4.1\times 10^{-50}$)	−0.330 ($5.9\times 10^{-8}$)	Myeloid
Breast	1384	+0.279 ($3.2\times 10^{-26}$)	−0.267 ($4.3\times 10^{-24}$)	−0.219 ($1.8\times 10^{-16}$)	Lymphoid
Lung adenoca.	860	+0.289 ($4.7\times 10^{-18}$)	−0.285 ($1.6\times 10^{-17}$)	−0.172 ($4.0\times 10^{-7}$)	Lymphoid
Colorectal	739	+0.191 ($1.6\times 10^{-7}$)	−0.175 ($1.8\times 10^{-6}$)	−0.189 ($2.3\times 10^{-7}$)	Lymphoid
Melanoma	701	+0.030 (0.510)	−0.028 (0.547)	−0.025 (0.581)	Independent

**Table 3:** Cox proportional-hazards diagnostics for the three models discussed in the text: breast cancer baseline, breast cancer immune-augmented, and pan-cancer stratified. The Schoenfeld column reports the minimum *p*-value across covariates in each model. The cross-validation concordance index is reported as mean ± standard deviation across five stratified folds, with the apparent concordance on the full sample given in parentheses for comparison. The DFBETA influential proportion is reported as the percentage of samples exceeding the $2/\sqrt{n}$ threshold for at least one covariate.

Model	*n*	Events	Schoenfeld Min *p*	CV *c*-Index (Apparent)
Breast baseline	1053	140	$3.2\times 10^{-4}$	0.720±0.085 (0.742)
Breast immune-augmented	1053	140	$1.8\times 10^{-4}$	0.720±0.074 (0.750)
Pan-cancer (stratified)	2295	584	$2.8\times 10^{-4}$	0.696±0.018 (0.699)

## Data Availability

All transcriptomic datasets analysed in this study are publicly available. Single-cell RNA sequencing data were obtained from the Gene Expression Omnibus under accession numbers GSE72056, GSE67835, GSE131928, GSE176078, GSE161529, GSE178341, and GSE131907. Bulk RNA sequencing data were obtained from the UCSC XENA TOIL Recompute distribution at https://xenabrowser.net/datapages/ (accessed on 25 May 2026). The STRING protein–protein interaction graph (version 12) is available at https://string-db.org/ (accessed on 25 May 2026). The LM22 immune cell-type signature is distributed with the CIBERSORT software at https://cibersortx.stanford.edu/ (accessed on 25 May 2026).
